# The role of property rights in shaping the effectiveness of protected areas and resisting forest loss in the Yucatan Peninsula

**DOI:** 10.1371/journal.pone.0215820

**Published:** 2019-05-08

**Authors:** Daniela A. Miteva, Peter W. Ellis, Edward A. Ellis, Bronson W. Griscom

**Affiliations:** 1 Department of Agricultural, Environmental and Development Economics, The Ohio State University, Columbus, OH, United States of America; 2 Global Lands, The Nature Conservancy, Arlington, VA, United States of America; 3 Centro de Investigaciones Tropicales, Universidad Veracruzana, Xalapa, Veracruz, Mexico; Rutgers The State University of New Jersey, UNITED STATES

## Abstract

The impact of different types of land tenure in areas with high biodiversity and threats of deforestation remains poorly understood. We apply rigorous quasi-experimental methods and detailed geospatial data to assess the role of tenure regimes—communally held lands (specifically, ejidos), private property, and their impact on the effectiveness of protected areas, in reducing forest loss in a biodiversity hotspot- the Yucatan peninsula in Mexico. We find evidence that, while protected areas are effective on average, their impact depends on the underlying type of tenure regime and forest, proxied by biomass levels and biome. Protecting communally held land may reduce deforestation, specifically the loss of medium- and high-biomass forests, compared to forests under private property regimes. Our results have important policy implications for the conservation and climate change mitigation efforts on the Yucatan. However, the high variance in forest loss rates among ejidos indicates that other characteristics of ejidos may be central to understanding community-based forest conservation opportunities.

## Introduction

There has been a recent push in the literature towards rigorous impact evaluations of interventions, coupled with a strong emphasis on how performance varies by context, specifically the underlying tenure regimes that create different incentives for conservation and land use clearing (e.g., [1–5]). However, still very little is known about the heterogeneity of interventions based on the context (e.g., [2, 3, 5]). We address this gap by examining how a common conservation intervention—protected areas, has varying impacts depending on the underlying property rights and the profitability of forests. We focus on the Yucatan peninsula in Mexico, an area with high biodiversity and importance for climate change mitigation (www. http://theredddesk.org/countries/mexico. Accessed July 19, 2017), but also high threats of forest conversion (e.g.,[6]).

Despite Mexico’s importance for conservation and climate change mitigation, not much is known about the effectiveness of conservation and climate change mitigation efforts in the area. A handful of studies examine the effectiveness of Mexico’s protected areas using rigorous statistical methods that account for the non-random placement of the interventions. Honey-Roses et al [7,8] use quasi-experimental techniques to assess the effectiveness of a butterfly reserve in central Mexico. In a country-wide analysis, Blackman et al. [9] find no statistically significant overall impact of protection between 1993 to 2000, but significant heterogeneity within regions. Pfaff et al. [10] examine the effectiveness of Mexico’s protected areas between 2000 and 2005 and find an overall 3.2% decrease in deforestation. The authors ascribe the change in effectiveness from 1990s to a shift in the conservation politics and funding. Sims & Alix-Garcia [11] find that Mexico’s protected areas and payments for ecosystem services reduced forest loss on average by 20–25% between 2000 and 2012, despite differences in the impacts on livelihoods. However, no previous study controls for the type of property rights.

Previous studies have suggested that the performance of protected areas may be significantly hindered by the lack of funds and effective institutions especially in developing countries [9,10]. Where formal protection may not be possible or easily enforced, establishing community land tenure has been considered an alternative as a way to incentivize and empower local communities to manage forest resources sustainably (e.g., [12–14]). The reason is the increasing returns to scale when forestry is practiced on a larger area; with limited other employment opportunities, forestry can generate employment opportunities and income that can benefit the whole community (e.g., [15,16]). In these cases, theory suggests, communities have a strong incentive to maintain the forest stocks. Theory has suggested that collective management of natural resources can be successful as a conservation strategy in cases where communities have existed for a long time and have clear rules and membership, conflict resolution mechanisms, recognized rights to organize; where standing forests generate significant benefits; where the forest resource is clearly delineated and stationary; and where there is little migration, ([15, 17]). Conversely, in areas where these conditions do not hold, the creation of private property may be a way to conserve resources by incentivizing investment in the land and facilitating monitoring and enforcement (e.g., [18]).

Large-scale empirical evidence on the role of tenure in forest conservation in Mexico is still largely lacking. To address the role of community land tenure in preventing deforestation, previous work on Mexico has relied on case studies (e.g., [13, 14, 19, 20, 21]), focused on only a particular type of common property resource—ejidos ([5, 22]), or does not sufficiently account for endogenous placement of interventions like protected areas, (e.g., [13, 20, 23,24, 25]).

Using rigorous statistical techniques and large-scale data, we examine the role of property rights, specifically, their interactions with protected areas, in preserving forests and the carbon stored within. We consider formal protected areas, community forest tenure (specifically, ejidos), parceled ejidos—a form of private property established from dividing formal ejido land, and private property in reducing forest loss in the Yucatan peninsula in Mexico. Specifically, while controlling for differences in the location of different tenure regimes, which proxy for the deforestation threats and the ease of enforcement, we examine what tenure regimes are effective in preventing forest loss. In addition, differentiating between forests biomes and combining biomass data with deforestation data, we examine the impact of the different tenure regimes across different types of forest. To our knowledge, ours is the first study that uses statistically rigorous techniques and large-scale data to (1) examine the interactions between protected areas, communally held forests, and private forests and (2) quantify the causal impact of community land tenure (ejidos) relative to private property and parceled ejidos. It is also one of few studies to address the role of property rights in conservation using large-scale data and a rigorous empirical design ([2, 5, 26]).

Our results indicate that protected areas in the Yucatan are effective on average, but, unsurprisingly, their impact varies by tenure regime. On average, we do not find evidence that community tenure in the absence of protected areas reduced forest loss relative to private properties. However, controlling for differences in the location, we find that protected areas in ejidos may be more effective than protection in other types of tenure. Consistent with previous studies (e.g.,[20]), we do find evidence that in the absence of protected areas, parceling ejidos may increase the probability of forest loss. Further, we provide evidence how the impacts vary by the type of forest (dry vs. moist broadleaf forests; high vs. low-biomass forests). Given the Yucatan’s importance for biodiversity conservation and climate change mitigation, our results underscore the need to understand the mechanisms through which interventions interact on the ground, in order to design effective conservation and development policies in the region.

## Methods & data

### Study area

**Fig 1:**
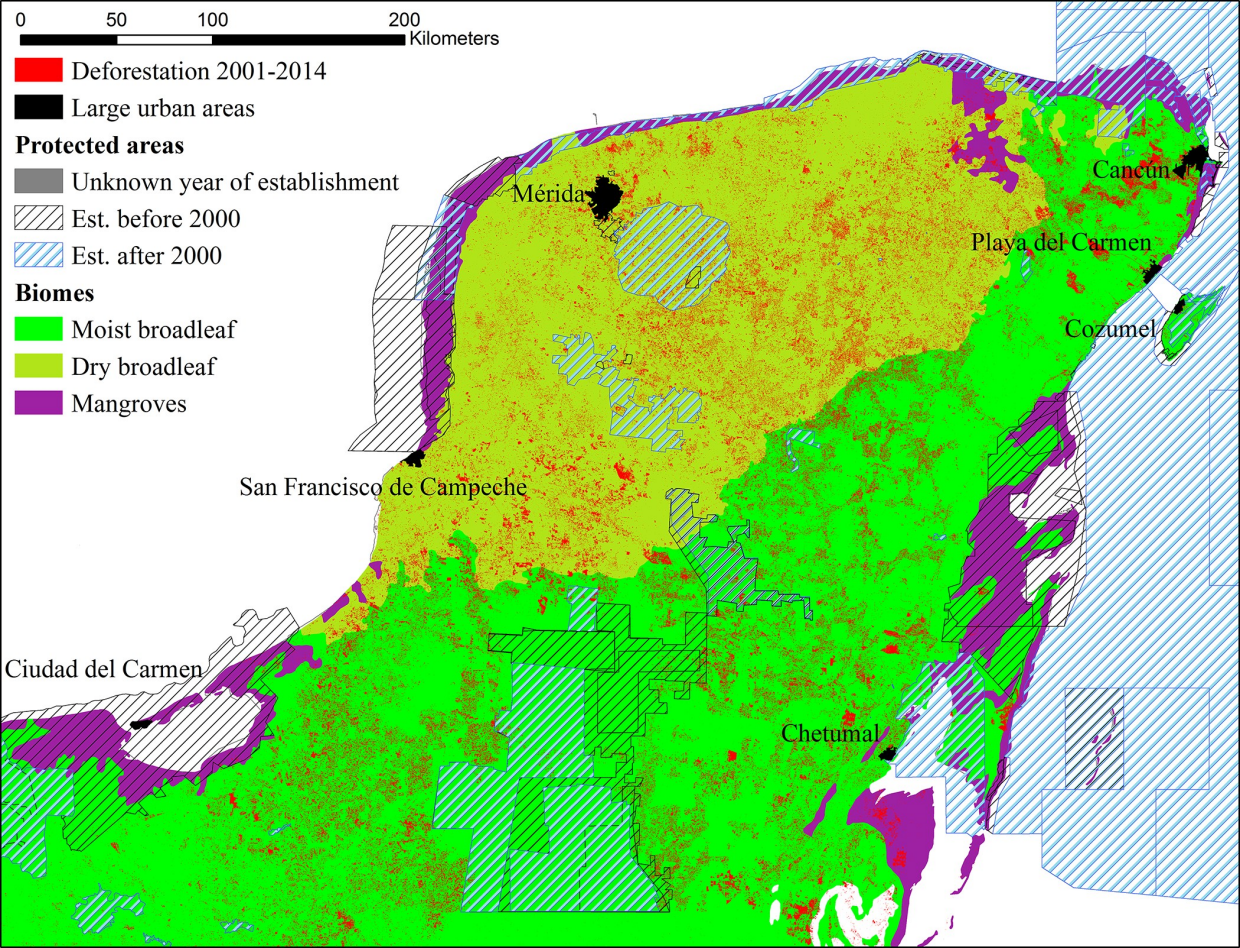
Total deforestation 2000–2014 by forest type in the three states (Yucatan, Quintana Roo, and Campeche), comprising our study area.

Our study area covers the three states in the Yucatan Peninsula: Campeche, Quintana Roo, and Yucatan. The area is considered a biodiversity hotspot; as part of the Maya Forest region, it contains the largest remnant of natural vegetation in Mesoamerica, high levels of species diversity and endemism, but also experiences high threats from human disturbance [27, 28]. We focus on the dry and moist broadleaf forests, which are the two forest biomes that span most of the peninsula (Fig 1) and store on average 311-533tC/ha and 455-755tC/ha, respectively (Table 1). They differ in their commercial importance: the dry broadleaf forests are converted to agricultural land, and harvested for fuelwood, marketable timber and charcoal, whereas the moist broadleaf forests are used more for commercial timber harvesting, but, to a smaller extent, also are cleared for agriculture and harvested for fuelwood and charcoal production [29]. Because of the variability of mangroves ecosystems comprised from shrub-like vegetation to trees [30], we exclude them from the analysis.

**Table 1:** Descriptive spatial statistics for tenure regimes and ecosystem types inside and outside protected areas (PA). The unprotected pieces of protected properties have been excluded from the calculations in this table.

**Dry broadleaf forests**				
**Category**	**Protection status**	**Tenure Regime**		
		**Ejido**	**Private property**	**Parceled ejido**
#properties	Inside Protected Area	51	171	117
	Outside Protected Area	2,094	9,745	5,593
area (ha)	Inside Protected Area	44,135.70	35,625.91	11,583.27
	Outside Protected Area	2,150,253.54	1,302,776.40	753,170.55
forest areas (ha)	Inside Protected Area	33,739.02	20,593.44	6,962.85
	Outside Protected Area	2,000,935.35	1,168,375.95	601,830.45
Mean biomass (tC/ha) Standard deviation in ()	Inside Protected Area	41.83 (19.91)	43.67 (23.77)	28.41 (19.07)
	Outside Protected Area	48.30 (20.02)	43.58 (24.89)	31.88 (23.32)
deforestation area (ha)	Inside Protected Area	1,399.59	433.98	958.86
	Outside Protected Area	232,098.93	144,424.17	85,655.34
%forest cleared 2000–2014	Inside Protected Area	4.15	2.11	13.77
	Outside Protected Area	11.60	12.36	14.23
% deforestation per year	Inside Protected Area	-0.003	-0.002	-0.01
	Outside Protected Area	-0.01	-0.01	-0.01
**Moist broadleaf forests**				
**Category**	**Protection status**	**Tenure Regime**		
		**Ejido**	**Private property**	**Parceled ejido**
#properties	Inside Protected Area	56	50	22
	Outside Protected Area	1,412	1,566	3,157
area (ha)	Inside Protected Area	550,695.84	23,778.04	9,315.35
	Outside Protected Area	3,283,911.27	498,309.62	759,727.83
forest areas in 2000 (ha)	Inside Protected Area	319,637.79	16,012.71	5,918.94
	Outside Protected Area	3,131,582.22	442,049.58	649,617.84
Mean biomass (tC/ha) Standard deviation in ()	Inside Protected Area	68.12 (20.64)	54.16 (24.90)	53.09 (18.35)
	Outside Protected Area	63.69 (19.39)	45.16 (25.80)	41.56 (26.17)
Total deforestation area (ha)	Inside Protected Area	9,259.47	707.40	174.69
	Outside Protected Area	352,922.49	64,877.49	114,171.39
% forest cleared 2000–2014	Inside Protected Area	2.90	4.42	2.95
	Outside Protected Area	0.11	0.15	0.18
% deforestation per year	Inside Protected Area	-0.002	-0.003	-0.002
	Outside Protected Area	-0.01	-0.01	-0.01

Forest loss on the peninsula is primarily driven by (1) conversion to pasture for commercial cattle ranching, maize cultivation and, more recently, large-scale mechanized agriculture for crops like soybeans, sugar cane, and sorghum [20, 31, 32, 33], (2) urban development especially in areas that are important for tourism or can accommodate waterfront properties [29, 32,34], (3) parcelization of ejidos, which can follow increases in the demand for urban, tourism, or agricultural land and/or can be the result of informal land markets and local arrangements (e.g., [20, 32, 35]) (4) and the practice of the traditional for the area slash-and-burn subsistence agriculture (called *milpa*) that results in small temporary openings in the forest canopy that are planted with maize, beans, squash and other subsistence crops, harvested for up to three years, and then left to regenerate into secondary forest [29, 31, 33]. In the Yucatán Península, greater forest cover loss from agricultural conversion was recently reported in Campeche and Yucatán compared to Quintana Roo, although forest cover loss from disturbances such as fires and from urbanization in tourist areas was greater in Quintana Roo [29].

While timber harvesting is a very important source of revenue for some local communities [13, 35], forestry practices in the region usually do not directly result in long-term conversion [34]. However, small-scale, usually temporary, tree canopy loss from logging operations does occur: the creation of gaps when individual trees are felled, the clearing of forests to create log landings and in some cases patch cuts of up to 4000m^2^ that allow for the natural regeneration of shade-intolerant species like mahogany [29, 36].

**Fig 2:**
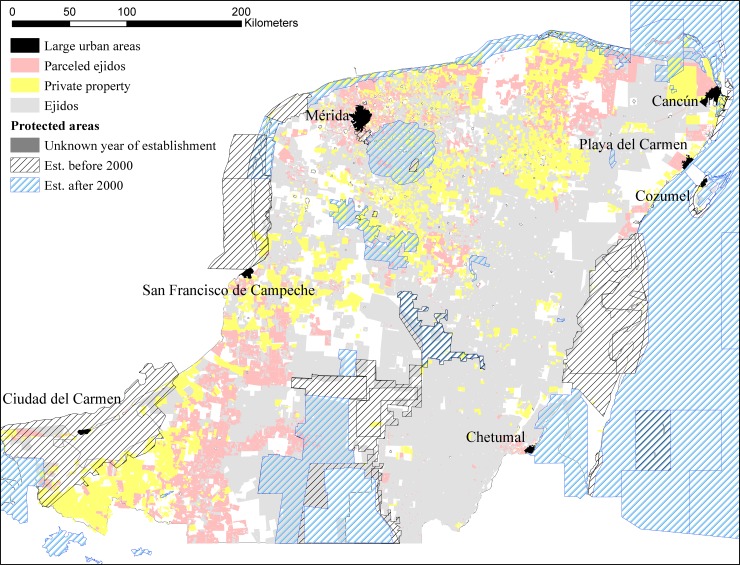
Distribution of the land tenure types within our study area. The areas with missing tenure information are in white and urban areas-in black.

The main tenure regimes in the area consist of private properties; ejidos, which are a form of collective land ownership and management; and former ejidos whose land has been parceled and allocated to individuals (Table 1; Fig 2). Ejidos comprise most of the study area: 5.5 Mha distributed among 3,613 properties (Table 1). Private properties encompass 1.8 Mha in 11,532 polygons; parceled ejidos span 1.5 Mha, distributed across 8,889 properties. A large fraction of the properties was covered in forest in 2000 (Table 1).

Established in 2 waves (1934–1940 and 1960s-1990s), ejidos traditionally have forests under communal use and agricultural land parceled and allocated to individual households [29]. Only a portion of the people living within an ejido have rights over the use and benefits of ejido forest resources and are responsible for forest management (these individuals are known as *ejidatarios*); the remaining households do not have rights to the ejido forest and do not directly benefit from it, although they may receive indirect benefits in the form of bribes not to deforest or improved community infrastructure (as in [15]). In ejidos, communal forests are managed separately from agricultural and urban areas; each ejidatario has access and decision rights to their portion of the agricultural and urban area, while management decisions for communal forest areas are undertaken by the ejido general assembly, made up of all ejidatarios. Although land use conversion inside communal forest areas is not permitted by the ejido and national law, illegal deforestation can take place under weak monitoring and enforcement [15, 20, 35]. Private property has been present before the establishment of ejidos; private property owners are primarily engaged in agriculture, residential or commercial real estate, tourism, and to a lesser extent, commercial forestry, mostly for charcoal production. Some ejidos have recently been divided into individual landholdings (we refer to these as parceled ejidos), although these are mostly informal and often not legally recognized [20, 32]. Because parceled ejidos may be in a state of flux, with forest stocks transitioning from collectively to individually owned and managed, we consider as a them as a separate category. Our sample spans ejidos parceled between 1993 and 2012. Only a small portion of existing ejidos (11%), parceled ejidos (1%), and private lands (3%) fall within protected areas, which place additional constraints on forest conversion and land use practices. For example, the Agrarian Law (*Ley Agraria*) restricts ejidos in establishing settlements or urban areas within protected areas; agriculture and cattle raising activities are allowed if they comply with the applicable Secretariat of Environment and Natural Resources (SEMARNAT) regulations. However, protected area management plans and conservation strategies by the federal Commission for Natural Protected Areas (CONANP) and state environmental institutions intend to discourage deforestation in overlapping and contiguous properties [37, 38].

We use pixels as the unit of analysis. For the sake of brevity, we refer to sample units under protected areas within existing ejidos, private properties, and parceled ejidos as protected ejidos, protected private property, and protected parceled ejidos, respectively. Similarly, the sample units located outside protected areas as well as outside the boundaries of properties spanning protected areas within existing ejidos, private properties, and parceled ejidos are referred to as unprotected ejidos, unprotected private property, and unprotected parceled ejidos throughout the text.

The details for the empirical analysis as well as the data used are described in the Methods and Data sections below.

### Methods

Our analysis answers the following questions:

- What was the average impact of protection within our study area between 2000 and 2014?
- What was the average impact of protection in private properties, existing ejidos, and parceled ejidos between 2000 and 2014?
- Did ejidos enhance the impact of formal protected areas more than private properties and parceled ejidos?

Question A allows us to quantify the average causal impact of protected areas in reducing deforestation in the Yucatan, whereas Question B allows us to test for heterogeneity in the impacts of protection based on the underlying property rights. The latter comparisons involve protected and unprotected pixels falling *under the same* tenure regime. While these comparisons allow us to establish the average causal impact of formal protected areas within a tenure regime, they do not allow us to compare one tenure regime to another. The reason is that we cannot rule out that the differences of the different tenure regimes in effectiveness may be driven by differences in their location. Controlling for differences in location, Question C allows us to further examine how tenure regimes impact the effectiveness of protected areas. In that analysis we first compare observationally similar *protected* pixels under one tenure regime to *protected* pixels under another tenure regime and then observationally similar *unprotected* pixels under one tenure regime to *unprotected* pixels under another. We also examine how the emerging patterns vary by the type of forest.

#### The need for matching techniques

Characteristics of the land like the biophysical attributes and proximity to markets and infrastructure determine its suitability and profitability for agriculture and development. Because forests experience different conversion threats as a function of their characteristics (e.g., how suitable the soil is for agriculture, how accessible timber markets are etc), not controlling for the differences in those is likely to invalidate causal inference (e.g., [2, 39, 40]). That is, simple comparisons of deforestation in the different tenure regimes will not allow us to attribute how much of that is due to the tenure regime itself and how much-to the location of the sample unit (e.g.,[2]).

Depending on the question, we use the presence of a formal protected area or a tenure regime as the treatment and control for its endogenous placement. In order to quantify the *causal* impact of the treatment on deforestation, we use matching techniques, which are a quasi-experimental statistical approach that allows us to establish what would have happened to a sample unit, had it not been placed under a given tenure regime (e.g., [41, 42]). The relevant statistical measure is the Average Treatment Effect on the Treated (ATT), with negative values indicating a reduction in the probability of forest loss due to the treatment. The choice of matching estimator for each specification is driven by the data: we use the matching estimator that results in the smallest covariate difference (as indicated by a normalized difference between the treatment and control distributions for that covariate) between the treatment and control groups. For the most part, we calculate the statistic using nearest neighbor matching with replacement, a Mahalanobis distance metric and trimming based on a propensity score [43]. The propensity score is the linearized predicted probability of an observation being under a particular treatment and is derived from a logit model that has the binary treatment as the outcome and biophysical and physiogeographic covariates related to the placement of the treatment and drivers of deforestation as independent variables. In some of the specifications, we use propensity-score augmented matching [44]. The type of matching for each specification used is reported in the Results summary tables. In all specifications, matching was done within a forest biome. Post-matching, we performed bias and variance adjustments [45, 46].

To examine the average impact of protection on deforestation (Question A above), we use as treatment pixels falling under a protected area, regardless of the tenure regime, and match them to observationally similar unprotected pixels, regardless of the tenure regime. In order to examine the average impact of protection in private properties, existing ejidos, and parceled ejidos (Question B), we perform matching within each tenure type, such that pixels falling under a protected area were considered as treatment and matched to observationally similar unprotected pixels from the same tenure regime, but not from the same property. Using analogous matching methods, we separately test for the possibility of spillovers resulting from displacing deforestation from the protected to the unprotected portions of a property. That is, for these specifications we consider the pixels from the unprotected pieces of properties partially spanned by a protected area as treatment and compare them to observationally similar pixels from properties not spanned by a protected area. We perform these tests for each tenure regime separately. Lastly, by controlling for the differences in the location, we compare each type of regime in terms of reducing deforestation (Question C). Specifically, in order to control for differences in the location of private properties and ejidos, we directly compare pixels in existing ejidos to observationally similar pixels under private property; we repeat the matching analysis for the pixels under parceled ejidos and private property, respectively. We perform the analysis for the protected and unprotected pixels, separately. That is, we compare forest loss in protected ejido pixels to observationally similar protected pixels in private property and forest loss in unprotected ejido pixels to observationally similar unprotected pixels in private property. We perform analogous tests comparing private properties and parceled ejidos. In all tests, we treat the two forest types separately.

In order to examine how the impacts of formal protected areas and the three tenure regimes vary with the type of forest (old-growth vs. not), we run partial linear models (PLM) using on each of the matched samples difference weights [47, 48]. Specifically, we run the PLM on an equation of the form:

$${tt}_{i}=\alpha +x_{i}\beta +f(z)+\epsilon_{i},$$

where *tt* is the treatment effect calculated for individual pair *i* (calculated as the difference between the outcome for the treated observation and its matched control), α is a constant, *ϵ_i_* is an idiosyncratic error term, *x_i_*-a vector of control variables, and *β*-a vector of the regression coefficients for each linear covariate. Motivated by the context in the Yucatan, the linear control variables include the distances to inland water, urban areas, roads (paved, unpaved, and highways), temperature, elevation, slope, precipitation, forest cover in 2000, and population density. z is the non-linear covariate—the amount of woody biomass in 2000, which proxies for old-growth vs. new forests. We generated 95% confidence intervals for the non-linear covariate via wild t bootstraps on 50 runs. The number of iterations was limited by the sample size and the resulting computational burdens.

Apart from allowing us to examine the impact on forests that differ in their importance for climate change mitigation and biodiversity, the PLM analysis addresses concerns that our estimates may be picking up temporary forest conversion due to shifting agriculture (e.g., *milpa*). Thus, we are able to examine whether forest loss is driven by clearing of lower biomass forests as part of traditional *milpa* agriculture.

### Data

Our source data include information on the boundaries of existing ejido lands, private property, parceled ejidos, national and federal terrains (Fig 2), formal protected areas, tree cover loss, and biophysical, socioeconomic, and physiographic covariates (Table 2). In cases of overlap, property boundaries were manually adjusted using an established topological rule-set. Because of data unavailability, we do not differentiate between different types of ejidos (e.g., forestry, urban, or agricultural) and instead consider them a single category; we address this limitation of our analysis in the Discussion section.

**Table 2:** Data sources and variable definitions.

Variable	Definition	Source
Tenure regimes	Boundaries for private properties, ejidos, and parceled ejidos	Existing and parceled ejidos: Registro Agrario Nacional. 2017. Tenencia de la tierra en México. México; Private property: Registro Agrario nacional. 2012. Tenencia de la tierra de México. México. Available at: https://datos.gob.mx/
Formal protected areas		World Database of Protected Areas 2015. Available at: https://www.protectedplanet.net
Forest loss 2000–2015	Binary variable, with 0 indicating no forest loss and 1- forest loss	Hansen et al. 2013. Available at: https://earthenginepartners.appspot.com/science-2013-global-forest/download_v1.2.html
**Covariates**		
Distance to inland water, in km	Euclidean distance	Environmental Systems Research Institute (ESRI). Available: https://www.arcgis.com/home/item.html?id=e750071279bf450cbd510454a80f2e63.
Distance to any urban area, in km	Euclidean distance	Based on census polygons
Distance to large urban areas, in km	Euclidean distance	Based on census polygons
Distance to large federal roads, in km	4-lane free paved roads	internal TNC database. Data available upon request
Distance to paved roads, in km	Euclidean distance	internal TNC database. Data available upon request
Distance to unpaved roads, in km	Euclidean distance	internal TNC database. Data available upon request
Distance to ports, in km	Euclidean distance	World Port Index Available at: http://msi.nga.mil/NGAPortal/MSI.portal?_nfpb=true&_pageLabel=msi_portal_page_62&pubCode=0015)
Temperature, in deg. C	Mean annual temperature	Instituto Nacional de Estadística y Geografía (INEGI). Available here: http://en.www.inegi.org.mx/app/mapa/espacioydatos/
Precipitation, in mm	Cumulative annual	Instituto Nacional de Estadística y Geografía (INEGI). Available here: http://en.www.inegi.org.mx/app/mapa/espacioydatos/
Elevation, in m	Based on 15m DEM	Instituto Nacional de Estadística y Geografía (INEGI). Available here: http://en.www.inegi.org.mx/app/mapa/espacioydatoshttp://en.www.inegi.org.mx/app/mapa/espacioydatos/
Slope, in deg.	15 m resolution	Based on the elevation layer
Biomass in 2000, tC	30 m resolution	Woods Hole 2015 Alianza MREDD+, 2013 (Alianza MREDD+, 2013. Mapa y base de datos sobre la distribución de la biomasa aérea de la vegetación leñosa en México. Versión 1.0. Woods Hole Research Center, USAID, CONAFOR, CONABIO, Proyecto México Noruega. México. Abril 2013. URL: http://www.alianza-mredd.org/componentes/monitoreo-reporte-y-verificacion/productos/mapa-de-la-densidad-de-carbono-en-biomasa-lenosa-aerea-de-los-bosques-y-selvas-en-mexico-2#.V4_JJ0aAOkq)
%Forest cover in 2000	30 m resolution	Hansen et al. 2013. Available at: https://earthenginepartners.appspot.com/science-2013-global-forest/download_v1.2.html
Population density in 2000	Spatially allocated population/km^2^	Columbia CIESIN (Available at: http://sedac.ciesin.columbia.edu/data/collections/browse)
Dominant vegetation type	Dry and moist broadleaf forests	WWF eco-zones layer. Available at: https://www.worldwildlife.org/publications/terrestrial-ecoregions-of-the-world

The formal protected area boundaries are based on the World Database on Protected Areas boundaries (Available at http://www.protectedplanet.net/. Accessed August 2018). Polygons listed as designated or inscribed and established before 2000 were included in the study. There are 63 designated protected areas on the peninsula under four different governance schemes (S1 Table); from those, we excluded lands within protected areas established after 2000, as newer protected areas may require more time to effect change. Our analysis does not distinguish between the different types of protected areas.

#### Sampling

Using a 30-meter pixel from the forest cover data as our unit of analysis, we randomly sample one percent of all areas forested in 2000; this resulted in ~1.6 million sample pixels; the sample size is constrained by the computational burden of very large datasets. We exclude all pixels that fell within urban locations and water bodies. To minimize the possibility of spatial dependence, we exclude spatially adjacent pixels (i.e., within 30 meters of another observation). To control for the possibility of spillovers within the same property, we exclude pixels that fall within the unprotected parts of protected tenure polygons; we use them to test whether protection displaces the forest clearing activities to the unprotected pieces of the properties (leakage or spillovers). An underlying assumption of the analysis is that spillovers from the protection of a property do not occur outside the property. Because of the small number polygons (n = 1) in the study area, we exclude all observations that are under indigenous lands or *comunidades*. While these also have common forest tenure regimes, they were formed by local, homogenous, indigenous groups (e.g., [49]). This is in contrast to ejidos that were created from often non-indigenous households from other regions and potentially different ethnic backgrounds. We further exclude observations from (a) two ejidos in Quintana Roo with current sustainable forest management certification (Noh Bec and Caoba, both of which obtained their Forest Sustainability Council (FSC) management certification in 2005), because of the changes in their governance, forestry practices, and access to financing that came with certification [50]; and (b) four Quintana Roo ejidos that had their forest sustainability management certification revoked during the study period. The reason is that these areas were very badly affected by Hurricane Dean in 2007, which damaged huge swaths of land and necessitated clear cutting of the damaged forests [50].

#### Covariates

Our statistical approach aims to establish what would have happened to a sample unit, had it not been placed under a treatment. To this end, we need to account for the characteristics of the sample units that would make them suitable for alternative units (e.g., agriculture, tourism). The covariates we use reflect the ease of deforestation and the suitability of the sample units for alternative land uses. We focus on available data representing the biophysical, physiogeographic, and socio-economic characteristics that affect profitability of wood products and charcoal (e.g., type of forest, proximity to markets proxied by distances to urban areas and ports), the suitability for agriculture (slope, elevation, precipitation, temperature, the proximity to water bodies for irrigation, and accessibility, proxied by the distance to roads, ports, and urban areas), and the suitability for tourism (accessibility, proxied by proximity to roads and urban cities as well as the proximity to water bodies for the potential for waterfront properties) (Table 2). In addition, we use the woody biomass density in 2000, to proxy for the type of forest—old growth vs. not, which, in turn, correlates with ease of conversion to agriculture and the profitability of timber. Further, we include population density in 2000, in order to proxy for the degree of urbanization. To capture differences in travel cost, we distinguish between three road types: paved, unpaved, and large federal roads connecting airports. We also differentiate large urban areas (Merida, San Francisco de Campeche, Cozumel, Chetumal, Cancun, and Playa de Carmen) from smaller towns, as large tourist centers likely influence outcomes differently (e.g., by creating more incentives for forest conversion to urban and tourist areas) than smaller locales that are more dependent on local agricultural labor markets. The choice of covariates, determined by data availability, is consistent with previous studies [13, 23, 51, 52, 53]. The covariates are calculated at the pixel level, which is our unit of analysis. The summary statistics are given in S3–S26 Tables.

#### Outcome

We focus on the aggregate forest loss between 2000 and 2014 as our metric for conservation performance. The data come from a global dataset at 30 meter resolution [54]. Several reasons underlie our choice of the Hansen data: First, they seem to be the dataset with finest resolution currently available for our study area. To the best of our knowledge, all other currently available datasets are at much coarser resolution (e.g., roughly 250-meters). Second, the methods behind Hansen dataset, including empirical uncertainty analysis and validation steps, are published and have been subjected to independent peer review, as part of the publication in high impact journal; the transparency sets the dataset apart from national datasets, for which methods and uncertainty assessments are often less accessible. Third, the Hansen dataset is currently one of the most widely used global dataset monitoring tree cover loss; its accessibility and widespread allows for comparisons across locations.

Following Mexico’s national forest definition [55], we consider pixels as forested, if the percent tree cover in 2000 is greater than 10% [55]. A pixel is counted towards forest loss if it was forested in 2000 and the forest was cleared between 2000 and 2014; pixels that were forested in 2000 and- remained forested in 2014 were considered no forest loss; pixels with no forest in 2000 are excluded from the analysis.

The choice of cutoff to define tropical forest is still subject to debate in the literature (e.g., [56]). The concern is that a low cutoff may be capturing tree loss in areas that are not forests (e.g., a mixture of herbaceous and tree crops). To address this issue, we supplement the deforestation data with the spatially explicit woody biomass data for 2000 that are consistent with the Hansen dataset [57]. Areas with trees that are not part of a forest have low values for woody biomass in 2000. Thus, by using partial linear regressions to examine the impacts of the interventions along a biomass gradient, we assess the sensitivity of our results to the choice of cutoff. We also repeated the analysis using 25% cutoff to define forest in 2000; the results are consistent with Tables 3–5 and available upon request.

**Table 3:** Average effectiveness of protected areas by forest type proxied by the average treatment effect on the treated (ATT). The observations falling within protected areas are labeled *protected* (or treated); the valid matched control -*unprotected*. The standard errors are given in parentheses and confidence intervals corresponding to each significance level—in square brackets. **n**_**t**_**, n**_**mc**_**, n**_**cp**_ indicate treated (on support), matched control, and control pool observations, respectively. A negative sign of the ATT indicates that protection reduced the probability of forest loss.

Ecosystem type	Mean protected	Mean unprotected	Raw ATT	Bias adj. ATT (std errors)	Observations (n_t_, n_mc_, n_cp)_	Matching
Dry broadleaf	0.04	0.08	-0.04*	-0.04*	7,140; 1,142; 446,655	Propensity score augmented, no corrections for the standard errors
			(0.02)	(0.02)		
			[-0.07; -0.01]	[-0.07; -0.01]		
Moist broadleaf	0.02	0.08	-0.07***	-0.07***	133,305;7,664; 528,749	Mahalanobis matching with trimming based on the propensity score
			(0.02)	(0.01)		
			[-0.12; -0.02]	[-0.10; -0.04]		

Significance levels

***1%

**5%

*10%.

**Table 4:** Average direct impacts of protection by tenure regime, proxied by the average treatment effect on the treated (ATT). The observations falling within protected areas (treatment) are compared to observationally similar unprotected pixels (control) in properties not intersecting a protected area. The standard errors are given in parentheses and confidence intervals corresponding to each significance level—in square brackets. **n**_**t**_**, n**_**mc**_**, n**_**cp**_ indicate treated (on support), matched control, and control pool observations, respectively. A negative sign of the ATT indicates that protection reduced the probability of forest loss.

Tenure regime	Forest type	Mean Treated	Mean Control	Raw ATT	Bias Adj. ATT	Sample (n_t_, n_c_, n_pool_)	Matching procedure
Ejidos	Dry	0.06	0.12	NA	NA	3,001, NA, 195,256	NA: very unbalanced covariate distributions
	Moist	0.03	0.13	-0.09***	-0.09***	29,435; 3,498; 318,826	Mahalanobis matching with trimming on the propensity score; Heteroscedasticity corrections for the standard errors
				(0.02)	(0.02)		
				[-0.14; -0.04]	[-0.14; -0.04]		
Private property	Dry	0.03	0.07	-0.04	-0.09***	1,438; 286; 104,426	Mahalanobis matching with trimming on the propensity score; Heteroscedasticity corrections for the standard errors
				(0.04)	(0.01)		
				[-0.11; 0.03]	[-0.12; -0.06]		
	Moist	0.03	0.03	0.01	-0.01	3,067; 423; 49,985	Propensity score augmented, no corrections for the standard errors
				(0.03)	(0.03)		
				[-0.04; 0.06]	[-0.06; 0.04]		
Parceled	Dry	0.03	0.11	NA	NA	32; NA; 6,967	NA: very small treated pool
	Moist	0.01	0.06	-0.05	-0.05**	396; 73; 6,127	Mahalanobis matching with trimming on the propensity score; Heteroscedasticity corrections for the standard errors
				(0.06)	(0.03)		
				[-0.15; 0.05]	[-0.10; -0.0002]		

Significance levels

***1%

**5%

*10%.

**Table 5:** Average spillover effects from protection proxied by the average treatment effect on the treated (ATT). The ATT captures the probability of forest loss in the unprotected portions of properties due to protection. In this case, a pixel is considered treated if it is within the unprotected portion of a protected property; the control group comprises of pixels located in fully unprotected properties. The standard errors are given in parentheses and confidence intervals corresponding to each significance level—in square brackets. n_t_, n_mc_, n_cp_ indicate treated (on support), matched control, and control pool observations, respectively. A negative sign of the ATT indicates that protection reduced the probability of forest loss.

Tenure regime	Forest type	Mean Treated	Mean Control	Raw ATT	Bias Adj. ATT	Sample (nt, nc, npool)	Matching procedure
Ejidos	Dry	0.07	0.10	-0.02***	-0.03***	33,357; 21,124; 182,568	Propensity score augmented, no corrections for the standard errors
				(0.01)	(0.01)		
				[-0.05; -0.01]	[-0.06; -0.004]		
	Moist	0.09	0.10	-0.01	-0.006	113,343; 17,504; 313,758	Mahalanobis matching with trimming based on the propensity score; heteroscedasticity corrections for the standard errors
				(0.01)	(0.01)		
				[-0.03; 0.007]	[-0.02; 0.11]		
Private property	Dry	0.05	0.09	-0.05***	-0.04***	848; 705; 104,310	Propensity score augmented, no corrections for the standard errors
				(0.02)	(0.02)		
				[-0.09; -0.003]	[-0.09; -0.001]		
	Moist	0.12	0.07	0.05***	0.05***	3,473; 2,531; 50,685	Propensity score augmented, no corrections for the standard errors
				(0.01)	(0.01)		
				[0.02; 0.08]	[0.02; 0.08]		
Parceled	Dry	0.11	0.14	-0.03***	-0.03***	4,026; 3,338; 52,478	Propensity score augmented, no corrections for the standard errors
				(0.01)	(0.01)		
				[-0.06; -0.004]	[-0.06; -0.004]		
	Moist	0.14	0.17	-0.03***	-0.03***	13,872; 8,638; 52,820	Propensity score augmented, no corrections for the standard errors
				(0.01)	(0.01)		
				[-0.06; -0.004]	[-0.06; -0.004]		

Significance levels

***1%

**5%

*10%

The ATT estimate gives us what would have happened to a treatment unit, had it not been placed under a treatment. In order to convert changes in the probability of deforestation to avoided deforestation and carbon dioxide emissions, we use back-of-the-envelope calculations based on the definition of the ATT estimate. Specifically, for each calculation we use the bias-adjusted ATT estimates and the mean probability of deforestation for the matched control group (Tables 3–6) as well as its mean biomass levels (S3–S26 Tables). That is, we multiply the ATT estimates by the mean deforestation rates within the matched control group and the area (in ha) of the matched control pixels. We then multiply the avoided deforestation area by the mean biomass levels corresponding to a forest and tenure types. Given the random sampling of the study area (roughly one percent sampled), we extrapolate the estimates to the whole study area by multiplying by 100.

**Table 6:** Average impact of tenure regimes on deforestation, proxied by the average treatment effect on the treated (ATT). The standard errors are given in parentheses and confidence intervals corresponding to each significance level—in square brackets. **n**_**t**_**, n**_**mc**_**, n**_**cp**_ indicate treated (on support), matched control, and control pool observations, respectively. A negative sign of the ATT indicates that deforestation in ejidos decreased the probability of forest loss relative to private property.

Tenure regime comparisons	Forest type	Mean Treated	Mean Control	Raw ATT	Bias Adj. ATT	Sample (n_t_, n_c_, n_pool_)	Matching procedure
Protected Ejidos (treated) vs. Protected Private Property (control)	Dry	0.05	0.05	-0.004	-0.03**	2,851; 434; 2,101	Mahalanobis matching with trimming based on the propensity score; Heteroscedasticity corrections for the standard errors
				(0.02)	(0.01)		
				[-0.04; 0.03]	[-0.05; -0.01]		
	Moist	0.04	0.01	0.03*	-0.02	26,145; 1,197; 3,228	Propensity score augmented, no corrections for the standard errors
				(0.01)	(0.01)		
				[0.004; 0.05]	[-0.002; 0.04]		
Protected ejidos (treated) vs. protected parceled (control)	Dry	0.10	0.11	-0.002	-0.02	851; 261; 365	Propensity score augmented, no corrections for the standard errors
				(0.03)	(0.03)		
				[-0.05; 0.05]	[-0.07; 0.03]		
	Moist	0.03	0.02	0.01	0.05	20,666; 733; 1,556	Propensity score augmented, no corrections for the standard errors
				(0.03)	(0.03)		
				[-0.05; 0.07]	[-0.004; 0.11]		
Protected private property (treated) vs. protected parceled (control)	Dry	0.10	0.03	NA	NA	2,101; NA, 32	Matching not feasible due to small control pool
	Moist	0.03	0.0003	0.03*	0.03*	3,067; 144; 424	Propensity score augmented, no corrections for the standard errors
				(0.02)	(0.02)		
				[0.004; 0.05]	[0.005; 0.05]		
Unprotected ejidos (treated) vs. unprotected private property (control)	Dry	0.13	0.13	0.002	0.004	174,944; 38,067; 104,426	Mahalanobis matching with trimming based on the propensity score
				(0.004)	(0.004)		
				[-0.01; 0.01]	[-0.003; 0.01]		
	Moist	0.12	0.10	0.02***	0.02***	298,411; 25,187; 50,039	Propensity score augmented, no corrections for the standard errors; result not robust
				(0.004)	(0.004)		
				[0.01; 0.03]	[0.01; 0.03]		
Unprotected ejidos (treated) vs. unprotected parceled (control)	Dry	0.13	0.13	-0.001	-0.005	174,994; 36,109; 50,081	Propensity score augmented, no corrections for the standard errors
				(0.003)	(0.003)		
				[-0.006; 0.004]	[-0.01; 0.0001]		
	Moist	0.12	0.16	-0.04	-0.04	298,407; 24,099; 52,851	Propensity score augmented, no corrections for the standard errors
				(0.02)	(0.02)		
				[-0.07; 0.001]	[-0.07; 0.0003]		
Unprotected private property (treated) vs. unprotected parceled (control)	Dry	0.13	0.11	0.03***	0.02***	107,537; 6,084; 6,948	Propensity score augmented, no corrections for the standard errors
				(0.01)	(0.01)		
				[0.004; 0.05]	[0.001; 0.05]		
	Moist	0.14	0.15	-0.01	-0.04**	54,562; 4,508; 5,605	Propensity score augmented, no corrections for the standard errors
				(0.02)	(0.02)		
				[-0.05; 0.03]	[-0.08; -0.01]		

Significance levels

***1%

**5%

*10%

## Results

### Protected areas were effective in protecting broadleaf forests between 2000 and 2014

**Fig 3:**
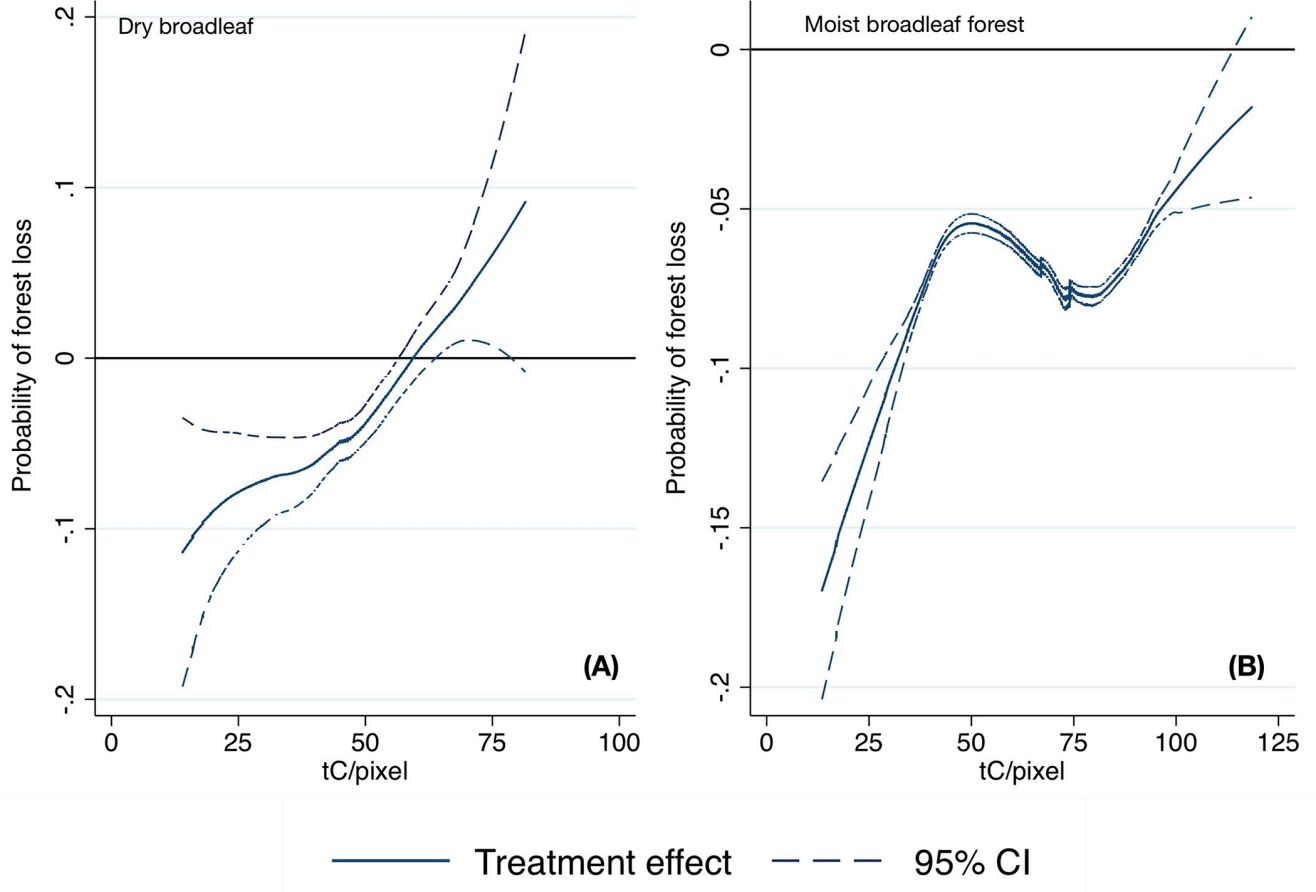
Results from the post-matching partial linear models comparing pixels under protected areas (treatment) to observationally similar non-protected pixels regardless of tenure for (A) dry broadleaf forests, and (B) moist broadleaf forests. Negative values indicate that a treatment (presence of a protected area) was effective in reducing forest loss for a given baseline biomass value; the estimate is statistically significant if the confidence intervals do not span the 0 horizontal line.

On average, protected areas reduced the probability of forest loss for dry and moist broadleaf forests by 4% and 7%, respectively (Table 3). Using back-of-the-envelope calculations for the whole study area, these correspond to over 400ha and 4,100 ha of avoided forest loss and 20.9 MtC and 282.3 MtC of avoided carbon emissions over 14 years, respectively. However, the impact of protection varied by the amount of biomass in 2000 (Fig 3). Protected areas were effective at protecting moist broadleaf forests except at very high biomass levels. For dry broadleaf forests, protection had a statistically significant impact only until intermediate biomass levels. These results suggest that across all tenure types formal protection may not be a deterrent for clearing high-biomass forests with high timber value, but can be effective when the forests may not have a very large commercial value.

### The protected area effectiveness between 2000 and 2014 varied by tenure regime

In private properties with dry broadleaf forests and existing ejidos with moist broadleaf forests, formal protection reduced the probability of forest loss by 9% between 2000 and 2014 (Table 4). Using extrapolations of the whole study area, these correspond to about 231.7 ha (12.36 MtC of avoided emissions) and 3,148 ha of avoided forest loss (218.6 MtC of avoided emissions), respectively. In parceled ejidos with moist broadleaf formal protection reduced the probability of deforestation by 5%, which translates into about 32.9 ha and 1.66 MtC over the study period. Formal protected areas had no statistically significant average impacts elsewhere (Table 4). Because of the small number of protected pixels under parceled ejidos in dry broadleaf forests and the very different characteristics of the protected and unprotected pixels for existing ejidos in dry broadleaf forests, we are omitting the results for the two specifications.

**Fig 4:**
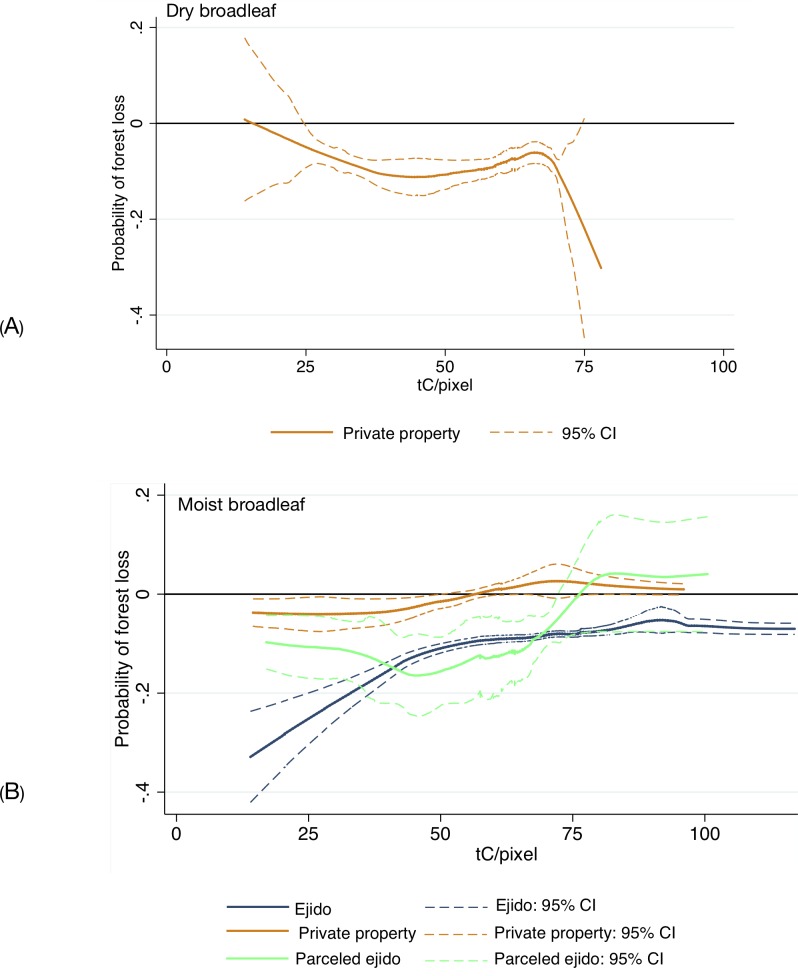
Results from the post-matching partial linear models comparing observationally similar protected and unprotected pixels under the 3 tenure regimes for (A) dry broadleaf forests (B) moist broadleaf forests. The negative values indicate that formal protection was effective in reducing forest loss relative to observationally similar pixel under the same tenure regime; the estimate is statistically significant if the confidence intervals do not span the 0 horizontal line. Because we could not find a viable specification for the ejidos and parceled ejidos in dry broadleaf forests, we do not provide estimates for those subsamples.

The post-matching heterogeneity analysis suggests that the impact of formal protected areas varied with the amount of biomass: in dry broadleaf forests, the protection of private property was generally effective except at very low biomass values (Fig 4A). In moist broadleaf forests, the impacts of protection was significant at low and intermediate values regardless of the tenure regime (Fig 4B). However, at high biomass levels, only the formal protection of ejidos remained a significant deterrent for deforestation. The impact of protecting ejidos in moist broadleaf forests is statistically significant regardless of the biomass levels, but is distinguishable from the other tenure regimes only at very low and very high levels of biomass.

Depending on the tenure regime, protection had different impacts on the unprotected forests in the same property (Table 5). In parceled ejidos in moist and dry broadleaf forests as well as existing ejidos and private properties in dry broadleaf forests, formal protection resulted in deforestation being reduced in the unprotected parts of the same properties. Based on back-of-the-envelope calculations, these translate into 2,332.3 ha (135.9 MtC), 901.3 ha (39.9 MtC), 5703.5ha (297.2 MtC), and 253.8ha (12.22MtC) of avoided forest loss (avoided carbon emissions) for the moist forest parceled ejidos, dry forest parceled ejidos, dry forest existing ejidos, and dry forest private properties, respectively. However, in private properties with moist broadleaf forests, protection seems to have displaced the deforestation activities to the unprotected parts of the protected properties, resulting in the loss of 1139ha (59.73 MtC). When the spillover effects are added to the direct impacts, the protection of private properties across the two forest types contributed to 653.5 ha being deforested (35.1 MtC of additional carbon emissions) due to leakage.

### Existing ejidos were more responsive to protection than private properties and parceled ejidos

**Fig 5:**
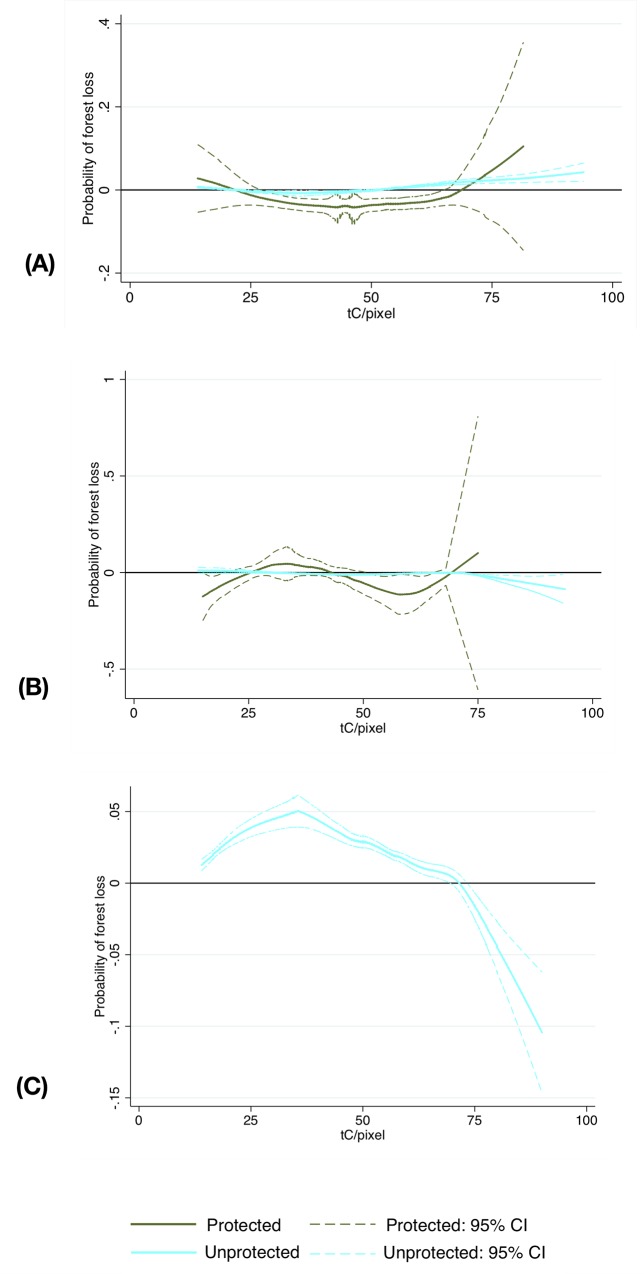
Results from the post-matching partial linear models comparing observationally similar pixels in dry broadleaf forests. Top panel: ejido (treatment group) compared to observationally similar private property pixels (control group); middle panel: ejido (treatment) compared to parceled ejido pixels (control); bottom panel: private property (treatment) compared to parceled ejido pixels (control). Negative values indicate that a treatment (pixels falling within protected and unprotected ejidos, respectively) was effective in reducing forest loss relative to observationally similar private properties; the estimate is statistically significant if the confidence intervals do not span the 0 horizontal line.

**Fig 6:**
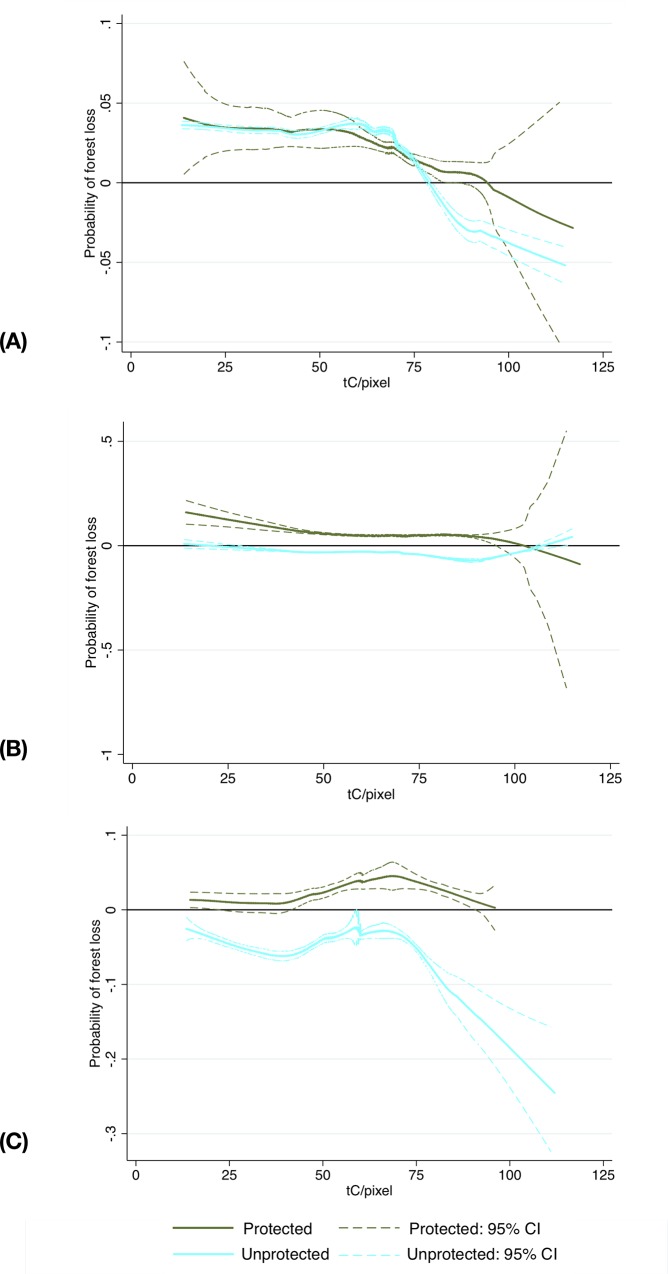
Results from the post-matching partial linear models comparing observationally similar pixels in moist broadleaf forests. Top panel: ejido (treatment group) compared to observationally similar private property pixels (control group); middle panel: ejido (treatment) compared to parceled ejido pixels (control); bottom panel: private property (treatment) compared to parceled ejido pixels (control). Negative values indicate that a treatment (pixels falling within protected and unprotected ejidos, respectively) was effective in reducing forest loss relative to observationally similar private properties; the estimate is statistically significant if the confidence intervals do not span the 0 horizontal line.

Existing ejidos, parceled ejidos, and private properties are located in different areas and may experience different deforestation threats (S6–S8 Tables). After controlling for the differences in location, the probability of dry broadleaf forest loss in unprotected ejidos was 0.13%, which is consistent with that for the observationally similar unprotected private properties (Table 6); in most broadleaf forests, the probability of forest loss for unprotected ejidos exceeded that of observationally similar private properties, with the difference being statistically significant. However, protection reversed the results: dry broadleaf forests in ejidos had 3% lower probability of being lost than in observationally similar private properties; moist broadleaf forests in existing ejidos had the same probability of deforestation as observationally similar private properties. The results from the post-matching heterogeneity analysis indicate that protected ejidos benefit dry and moist broadleaf forests at intermediate biomass levels relative to observationally similar pixels in private properties (Figs 5 & 6).

Parceled ejidos seem to have a mixed impact on forest conservation during the study period. Our results comparing pixels under parceled ejidos to observationally similar pixels under existing ejidos, indicate that, on average there were no statistically significant differences between the two tenure types for both dry and moist broadleaf forests (Table 6). In dry and moist broadleaf forests, unprotected ejidos had a lower probability of deforestation at medium to high levels of biomass relative to unprotected parceled ejidos (Figs 5 & 6). Relative to parceled ejidos, protection in existing ejidos reduced the probability of dry broadleaf forest loss only at intermediate biomass levels and increased it in moist broadleaf forests. A potential explanation for the observed pattern is that the protected areas in our sample may allow multiple use and controlled timber harvesting, which may be practiced by the existing ejidos only.

Unprotected parceled ejido pixels exhibited 2% lower and 4% higher probability of forest loss relative to observationally similar private property pixels in dry and moist broadleaf forests, respectively (Table 6). The post-matching PLM analysis indicates that much of the value in dry broadleaf forests is driven by impacts at low and intermediate biomass levels (Fig 5 Panel C); for moist broadleaf forests the effect did not vary with the biomass levels (Fig 6 Panel C). In contrast, moist broadleaf pixels in protected parceled ejidos had on average 3% lower probability of deforestation relative to observationally similar private property pixels, with the effect most pronounced at intermediate biomass levels (Fig 6 Panel C). The very small sample size does not allow us to estimate the impact of protected private property pixels relative to observationally similar protected parceled ejido pixels in dry broadleaf forests.

The ambiguous impact of parceled ejidos may very likely be due to differences in the timing of ejido parcelization as well as potential livelihood strategies, with recently parceled ejidos deforesting more to clear land for other activities as they transition to private properties and/or different livelihoods [20]. Our dataset indicating the boundaries of the parceled ejidos does not allow us to distinguish when the ejidos were divided.

## Discussion

Our study is the first to provide large-scale rigorous evidence of the impact of common and private forest tenure regimes and their interactions with protected areas in a biodiversity hotspot like the Yucatan peninsula. Our results indicate that protected areas in the Yucatan peninsula are generally an effective conservation strategy that can also contribute to climate change mitigation. Our estimates of conservation effectiveness of protected areas are smaller than those of previous studies (e.g., [10, 11]). There are two possible explanations for the observed patterns. First, we use a different forest loss data source than Pfaff et al. [10]; our cutoff to define forests is lower than the one used in Sims & Alix-Garcia [11], who also use a different sample unit (locality) instead of pixels, although robustness checks with higher cutoffs indicated similar patterns. Given the largely insignificant impacts of protection at low levels of biomass in our study area, a lower cutoff to define forests suggests that we are likely underestimating the impact of formal protected areas. Second, our analysis does not differentiate between the different types of protected areas. Most protected areas on the Yucatan peninsula are biosphere reserves (48,380km^2^), followed by mixed use (10,637km^2^) and strict protected areas (9,826.1km^2^). Of the private properties spanned by a protected area, 55% are under a mixed use, followed by biosphere reserves (31%) and strict protected areas (13%). Of the ejidos spanned by a protected area, 70% are under a biosphere reserve, 18% are under a mixed use, and 12% are under a strict protected area. While previous studies have suggested that the impact of protection might depend on the category of the protected area (e.g., [5, 10, 11]), we do not explicitly test for heterogeneity based on protected area category. The reasons are (1) the relatively small sample sizes in this study precluded us from explicitly testing this hypothesis and (2) digression from the scope of the article that aiming to establish the average impacts of protection interacted with tenure regimes and type of forest. Examining the interaction of the different types of protected areas and tenure regimes is an interesting venue for future research.

We find that ejidos may be a successful tool in promoting forest conservation and climate change mitigation in the Yucatan. Our estimates regarding ejidos relative to protected areas are conservative, i.e., biased towards finding no impacts. The reason is that ejidos may have voluntary conservation areas and community forestry outside the formal protected areas [34]; the delineation of such conservation zones is often not publicly available and not accounted for in the analysis. For this reason, if conservation zones that help maintain forest cover are more likely to exist outside formal protected areas (thereby effecting the control group), any additional protected area effect they create would diminish the effect of formal protected areas. That is, we would observe a smaller impact of the formal protected areas on average. Further, unprotected ejidos receiving payments to protect forests and provide ecosystem services as well as those receiving technical assistance for monitoring and enforcement from non-governmental organizations are also likely to have lower deforestation rates, although the effect may be short-lived [11, 58, 59]. Similarly, the exclusion of the ejidos under Forest Sustainability Council (FSC) certification implies that the ejidos that are likely to perform best in terms of forest management have been excluded from the analysis and is, therefore, biasing the estimates of ejido effectiveness towards 0. However, as of now, these are only a small number.

In contrast to previous studies that find that ejidos and protected areas have the same impact on forests (e.g., [59, 60]) or those that find that community forest tenure may reduce forest loss relative to private property in Mexico and elsewhere (e.g., [31, 61]), we find that broadleaf forests in ejidos *in the absence of conservation interventions*, may have a higher probability of being cleared than those in private properties. There are two potential explanations for the observed patterns. First, we consider the impact of ejidos *in aggregate*. That is, our analysis does not distinguish between forestry and non-forestry ejidos as well as those receiving additional support and assistance (e.g., payments for ecosystem services, support for silvicultural practices etc). Because the non-forestry ejidos do not depend on the forest as a primary source of livelihoods, we expect that forest loss there may be greater there (e.g., [20]). Second, our data do not allow us to distinguish whether forestry is practiced in private properties, and if so, what species are harvested and how. Thus, if the latter harvest species different from mahogany, for example, the logging may not register as deforestation in the satellite data used to quantify the deforestation outcome.

Because of data limitations, our analysis presents an average impact of ejidos on forests in the Yucatan. Other studies have shown that larger ejidos with greater numbers of ejidatarios are also associated with higher probabilities of forest loss, although these ejidos may also be effective in maintaining forest cover when permanent forest areas and silviculture are present [13, 59]. Furthermore, we do not account for the differences in the ejido size, area of remaining forest within ejidos, or tree species within the forest holdings, which impact the profitability of forests and, hence, the incentives to convert the land to other uses [20, 32]. Moreover, because large-scale data are not available, we do not account of the socio-economic characteristics of ejidos: factors such as low socio-economic heterogeneity within the ejido member group, lower numbers of ejidatarios, low poverty levels, high dependence on forests, longer history of forest use, history of cooperation, and indivisible well-delineated stationary resources have been associated with higher potential for collective action and better ability to enforce regulations and monitor forests. [7, 15, 21, 29, 59]. Finally, domestic and international policies can also undermine the effectiveness of forestry ejidos. For example, recent policy changes such as an increase in agricultural subsidies and more liberal trade that allow for surges in timber imports from China–these policy interventions can undermine the profitability of Mexico’s domestic forestry enterprises and create incentives for the conversion of the land to other uses [29]. The effects of these policies remain understudied.

The protected areas in ejidos appeared to have a net positive effect on moist broadleaf forests. Furthermore, the existing ejidos appeared more responsive to protection than private properties. Previous studies suggest this may be due to protection increasing tenure security and contributing to greater political and economic equality in the ejido, thus, incentivizing and facilitating ejidos to protect forests [62]. Because 60% of Mexico’s forests are currently managed by ejidos [63], it appears that strategic conservation efforts should target ejidos. In this context, three important venues for applied research emerge: (1) understanding how and why the forest ecosystems are changing in ejidos, (2) developing and testing mechanisms through which interventions in ejidos effect change on the ground, and (3) understanding the mechanisms to preserve ejidal forests as community managed and not as individual property. Similar to other studies relying on geospatial forest cover data, we do not account for changes in the composition of the forest ecosystems (such as the loss of mahogany), which may be changing in different ways due to differences in the forest management practices [60]. Because ejidos are dynamic systems [21], it is important to identify the most strategic incentives to minimize environmental impacts and conserve forest ecosystems, while maintaining rural livelihoods and improving human well-being. Future research is needed to inform the development of policies to achieve desirable reductions in forest loss and promote the sustainable development of the region. Consistent with previous studies [9, 11], we find that protected areas can be effective, with the impact varying based on location. These complex contingencies require well-designed conservation and development plans based on a thorough understanding of the land use decisions and socio-economic processes operating in different tenure regimes. Only then can decision makers adopt the combination of policies that are likely to be most effective in a given area.

## Supporting information

S1 Table Summary of the protected areas on the Yucatan Peninsula. Because protection may necessitate time to effect change, we dropped the protected areas established after 2000 from the analysis. We follow the classification in [11].
(DOCX)

S2 Table Variable codes and definitions for the covariate balance tables below. The following tables represent the covariate balances of the matched and unmatched samples for all of the comparisons. The normalized differences are calculated using the formula in [42] and a threshold of 0.25.
(DOCX)

S3 Table Protected vs. unprotected pixels regardless of tenure–dry broadleaf forests. (DOCX)

S4 Table Protected vs. unprotected pixels regardless of tenure–moist broadleaf forests. (DOCX)

S5 Table Protected vs. unprotected pixels within ejidos–moist broadleaf forests. (DOCX)

S6 Table Protected vs. unprotected pixels within private properties–dry broadleaf forests. (DOCX)

S7 Table Protected vs. unprotected pixels within private properties–moist broadleaf forests. (DOCX)

S8 Table Protected vs. unprotected pixels within parcelized ejidos–moist broadleaf forests. (DOCX)

S9 Table Covariate balance table for unprotected pixels within protected ejidos matched to observationally similar pixels in unprotected ejidos in dry broadleaf forests. (DOCX)

S10 Table Covariate balance table for unprotected pixels within protected ejidos matched to observationally similar pixels in unprotected ejidos in moist broadleaf forests. (DOCX)

S11 Table Covariate balance table for unprotected pixels within protected private properties matched to observationally similar pixels in unprotected private properties in dry broadleaf forests. (DOCX)

S12 Table Covariate balance table for unprotected pixels within protected private properties matched to observationally similar pixels in unprotected private properties in moist broadleaf forests. (DOCX)

S13 Table Covariate balance table for unprotected pixels within protected parceled ejidos matched to observationally similar pixels in unprotected parceled ejidos in dry broadleaf forests. (DOCX)

S14 Table Covariate balance table for unprotected pixels within protected parceled ejidos matched to observationally similar pixels in unprotected parceled ejidos in moist broadleaf forests. (DOCX)

S15 Table Covariate balance tables for protected ejido pixels matched to protected private property pixels—Dry broadleaf forest. (DOCX)

S16 Table Covariate balance tables for protected ejido pixels matched to protected private property pixels—Moist broadleaf forest. (DOCX)

S17 Table Covariate balance tables for protected ejido pixels matched to protected parceled ejido pixels—Dry broadleaf forest. (DOCX)

S18 Table Covariate balance tables for protected ejido pixels matched to protected parceled ejido pixels—Moist broadleaf forest. (DOCX)

S19 Table Covariate balance tables for protected private property pixels and protected parceled ejido pixels—Dry broadleaf forest. Because of the small control pool (n = 32), matching was not possible.
(DOCX)

S20 Table Covariate balance tables for protected private property pixels matched to protected parceled ejido pixels—Moist broadleaf forest. (DOCX)

S21 Table Covariate balance tables for unprotected ejido pixels matched to unprotected private property pixels—Dry broadleaf forest. (DOCX)

S22 Table Covariate balance tables for unprotected ejido pixels matched to unprotected private property pixels—Moist broadleaf forest. (DOCX)

S23 Table Covariate balance tables for unprotected ejido pixels matched to unprotected parceled ejido pixels—Dry broadleaf forest. (DOCX)

S24 Table Covariate balance tables for unprotected ejido pixels matched to unprotected parcelized ejido pixels—Moist broadleaf forest. (DOCX)

S25 Table Covariate balance tables for unprotected parcelized ejido pixels matched to unprotected private property pixels—Dry broadleaf forest. (DOCX)

S26 Table Covariate balance tables for unprotected parcelized ejido pixels matched to unprotected private property pixels—Moist broadleaf forest. (DOCX)
