# Supplementary material for: The role of property rights in shaping the effectiveness of protected areas and resisting forest loss in the Yucatan Peninsula
Source: PLoS One. 2019 May 8;14(5):e0215820. doi: 10.1371/journal.pone.0215820 (PMC6505956; doi:10.1371/journal.pone.0215820)
Supplement: S1 Table — Because protection may necessitate time to effect change, we dropped the protected areas established after 2000 from the analysis. We follow the classification in [11]. (DOCX) [file pone.0215820.s001.docx]

| **Protected Area Name** | **Designation** | **Classified as** | **Year established** | **Area (in km^2^)** |
| --- | --- | --- | --- | --- |
| Arrecifes de Sian Ka'an | Biosphere Reserve | Biosphere reserve | 1998 | 393.9 |
| Río(a) Lagartos | Biosphere Reserve | Biosphere reserve | 1999 | 697.1 |
| Río(a) Celestún | Biosphere Reserve | Biosphere reserve | 2000 | 930.2 |
| Banco Chinchorro | Biosphere Reserve | Biosphere reserve | 1996 | 1607.0 |
| Los Petenes | Biosphere Reserve | Biosphere reserve | 1999 | 3207.1 |
| Pantanos de Centla | Biosphere Reserve | Biosphere reserve | 1992 | 3358.2 |
| Los Petenes | Special Protection Zone of Aquatic Flora and Fauna | Biosphere reserve | 1996 | 4461.5 |
| Sian Ka'an | UNESCO-MAB Biosphere Reserve | biosphere reserve | 1986 | 5939.3 |
| Sian Ka'an | Biosphere Reserve | Biosphere reserve | 1986 | 5943.1 |
| Sian Ka'an | World Heritage Site | biosphere reserve | 1987 | 5943.1 |
| Región de Calakmul | UNESCO-MAB Biosphere Reserve | Biosphere reserve | 1993 | 7882.2 |
| Calakmul | Biosphere Reserve | Biosphere reserve | 1989 | 8017.4 |
| Kabah | Urban Park | mixed use | 1995 | 0.5 |
| Uaymil | Flora and Fauna Protection Area | Mixed use | 1994 | 996.0 |
| Yum Balam | Flora and Fauna Protection Area | Mixed use | 1994 | 1779.5 |
| Laguna de Términos | Flora and Fauna Protection Area | Mixed use | 1994 | 7861.0 |
| Laguna de Chancanaab | Natural Park | Strict PA | 1983 | 0.2 |
| Playa de la Isla Contoy | Sanctuary | Strict PA | 1986 | 0.2 |
| Santuario de la Tortuga Marina Xcacel - Xcacelito (Terrestre) | Area Subject to Ecological Conservation | Strict PA | 1998 | 0.2 |
| Playa Adyacente a la localidad denominada Río Lagartos | Sanctuary | Strict PA | 1986 | 1.5 |
| Laguna de Manati | Area Subject to Ecological Conservation | Strict PA | 1999 | 2.3 |
| Santuario de la Tortuga Marina Xcacel - Xcacelito (Marino) | Area Subject to Ecological Conservation | Strict PA | 1998 | 3.9 |
| Dzibilchantún | National Park | Strict PA | 1987 | 6.2 |
| Tulum | National Park | Strict PA | 1981 | 7.4 |
| Kabah | State Park | Strict PA | 1993 | 11.4 |
| San Juan Bautista Tabi y Anexa Zac Nicte | Natural Protected Area of scenic, historic and cultural value | Strict PA | 1994 | 16.2 |
| Refugio Estatal de Flora y Fauna Laguna Colombia | Area Subject to Ecological Conservation | Strict PA | 1999 | 17.5 |
| Refugio Estatal de Flora y Fauna Sistema Lagunar Chacmochuch | Area Subject to Ecological Conservation | Strict PA | 1999 | 22.0 |
| Isla Contoy | National Park | Strict PA | 1998 | 59.2 |
| Lagunas de Yalahau | State Park | Strict PA | 1999 | 62.3 |
| Costa Occidental de Isla Mujeres, Punta Cancún y Punta Nizuc | National Park | Strict PA | 1996 | 100.7 |
| Arrecife de Puerto Morelos | National Park | Strict PA | 1998 | 104.8 |
| Cuxtal | Area Subject to Ecological Conservation | Strict PA | 1993 | 120.1 |
| Arrecifes de Cozumel | National Park | Strict PA | 1996 | 157.4 |
| Arrecifes de Xcalak | National Park | Strict PA | 2000 | 199.5 |
| Dzilam (reserva estatal) | Ramsar Site, Wetland of International Importance | Strict PA | 2000 | 693.3 |
| Humedal de Importancia Especialmente para la Conservación de Aves Acu├íticas Reserve Ría Lagartos | Ramsar Site, Wetland of International Importance | Strict PA | 1986 | 693.8 |
| Balam-Kin | Area Subject to Ecological Conservation | Strict PA | 1999 | 1113.6 |
| Santuario del Manati, Bahia de Chetumal | Area Subject to Ecological Conservation | Strict PA | 1996 | 3094.7 |
| Reserva de la Biosfera Pantanos de Centla | Ramsar Site, Wetland of International Importance | Strict PA | 1995 | 3337.7 |
