# Supplementary material for: The role of property rights in shaping the effectiveness of protected areas and resisting forest loss in the Yucatan Peninsula
Source: PLoS One. 2019 May 8;14(5):e0215820. doi: 10.1371/journal.pone.0215820 (PMC6505956; doi:10.1371/journal.pone.0215820)
Supplement: S2 Table — The following tables represent the covariate balances of the matched and unmatched samples for all of the comparisons. The normalized differences are calculated using the formula in [42] and a threshold of 0.25. (DOCX) [file pone.0215820.s002.docx]

| **Variable code** | **Definition** |
| --- | --- |
| dist2inlandwater_km | Distance to inland water, in km |
| dist2any_urban_km | Distance to any urban area, in km |
| dist2largefedrd_km | Distance to large federal roads, in km |
| dist2largeurban_km | Distance to large urban, in km |
| dist2pavedrd_km | Distance to paved roads, in km |
| dist2port_km | Distance to ports, in km |
| dist2unpavedrd_km | Distance to unpaved roads, in km |
| temper | Temperature (in degrees) |
| biomass00 | Biomass in 2000 |
| elev_m | Elevation, in meters |
| forest00 | %Forest cover in 2000 |
| pop00 | Population in 2000 |
| slope_deg | Slope (in degrees) |
| precip | Precipitation (in mm) |
