# Supplementary material for: The role of property rights in shaping the effectiveness of protected areas and resisting forest loss in the Yucatan Peninsula
Source: PLoS One. 2019 May 8;14(5):e0215820. doi: 10.1371/journal.pone.0215820 (PMC6505956; doi:10.1371/journal.pone.0215820)
Supplement: S3 Table — (DOCX) [file pone.0215820.s003.docx]

| **Variable** | **Sample** | **Mean** | | **%bias** | **%reduct  \|bias\|** | **norm. diff** |
| --- | --- | --- | --- | --- | --- | --- |
|  |  | **Treated** | **Control** |  |  |  |
| dist2inlandwater_km | Unmatched | 13.02 | 41.08 | -183.00 |  | -1.29 |
|  | Matched | 13.02 | 16.29 | -21.40 | 88.30 | -0.15 |
| dist2any_urban_km | Unmatched | 23.58 | 12.27 | 97.40 |  | 0.69 |
|  | Matched | 23.58 | 22.01 | 13.60 | 86.10 | 0.10 |
| dist2largefedrd_km | Unmatched | 26.63 | 13.53 | 110.70 |  | 0.78 |
|  | Matched | 26.63 | 25.73 | 7.70 | 93.10 | 0.05 |
| dist2largeurban_km | Unmatched | 48.93 | 82.92 | -93.70 |  | -0.66 |
|  | Matched | 48.93 | 50.33 | -3.90 | 95.90 | -0.03 |
| dist2pavedrd_km | Unmatched | 10.62 | 5.17 | 85.70 |  | 0.61 |
|  | Matched | 10.62 | 9.79 | 13.10 | 84.70 | 0.09 |
| dist2port_km | Unmatched | 87.17 | 104.76 | -43.30 |  | -0.31 |
|  | Matched | 87.17 | 87.61 | -1.10 | 97.50 | -0.01 |
| dist2unpavedrd_km | Unmatched | 18.95 | 19.80 | -6.30 |  | -0.04 |
|  | Matched | 18.95 | 19.83 | -6.50 | -4.20 | -0.05 |
| temper | Unmatched | 25.92 | 25.92 | -0.60 |  | 0.00 |
|  | Matched | 25.92 | 25.90 | 7.20 | -1174.10 | 0.05 |
| biomass00 | Unmatched | 95.69 | 101.45 | -18.70 |  | -0.13 |
|  | Matched | 95.69 | 95.11 | 1.90 | 89.90 | 0.01 |
| elev_m | Unmatched | 7.78 | 38.75 | -107.80 |  | -0.76 |
|  | Matched | 7.78 | 9.23 | -5.10 | 95.30 | -0.04 |
| forest00 | Unmatched | 80.81 | 80.04 | 3.80 |  | 0.03 |
|  | Matched | 80.81 | 80.34 | 2.30 | 39.20 | 0.02 |
| pop00 | Unmatched | 98.91 | 32.81 | 36.80 |  | 0.26 |
|  | Matched | 98.91 | 100.57 | -0.90 | 97.50 | -0.01 |
| slope_deg | Unmatched | 0.17 | 0.94 | -48.10 |  | -0.34 |
|  | Matched | 0.17 | 0.12 | 3.00 | 93.70 | 0.02 |
| precip | Unmatched | 3005.70 | 2892.20 | 57.30 |  | 0.41 |
|  | Matched | 3005.70 | 2971.30 | 17.40 | 69.70 | 0.12 |
