# Supplementary material for: The role of property rights in shaping the effectiveness of protected areas and resisting forest loss in the Yucatan Peninsula
Source: PLoS One. 2019 May 8;14(5):e0215820. doi: 10.1371/journal.pone.0215820 (PMC6505956; doi:10.1371/journal.pone.0215820)
Supplement: S4 Table — (DOCX) [file pone.0215820.s004.docx]

| **Variable** | **Sample** | **Mean** | | **%bias** | **%reduct  \|bias\|** | **norm. diff**  **Treated** |
| --- | --- | --- | --- | --- | --- | --- |
|  |  | **Treated** | **Control** |  |  |  |
| dist2inlandwater_km | Unmatched | 30.73 | 18.53 | 77.60 |  | 0.55 |
|  | Matched | 30.73 | 29.21 | 9.60 | 87.60 | 0.07 |
| dist2any_urban_km | Unmatched | 46.49 | 28.63 | 95.80 |  | 0.68 |
|  | Matched | 46.49 | 41.18 | 28.50 | 70.30 | 0.20 |
| dist2largefedrd_km | Unmatched | 38.35 | 22.52 | 82.70 |  | 0.58 |
|  | Matched | 38.35 | 35.68 | 14.00 | 83.10 | 0.10 |
| dist2largeurban_km | Unmatched | 130.02 | 110.47 | 36.40 |  | 0.26 |
|  | Matched | 130.02 | 118.34 | 21.70 | 40.30 | 0.15 |
| dist2pavedrd_km | Unmatched | 17.12 | 11.18 | 57.90 |  | 0.41 |
|  | Matched | 17.12 | 14.36 | 27.00 | 53.40 | 0.19 |
| dist2port_km | Unmatched | 181.69 | 157.83 | 37.20 |  | 0.26 |
|  | Matched | 181.69 | 182.10 | -0.60 | 98.30 | 0.00 |
| dist2unpavedrd_km | Unmatched | 29.39 | 18.18 | 75.10 |  | 0.53 |
|  | Matched | 29.39 | 25.41 | 26.70 | 64.50 | 0.19 |
| temper | Unmatched | 26.03 | 26.04 | -2.00 |  | -0.01 |
|  | Matched | 26.03 | 26.04 | -1.80 | 9.00 | -0.01 |
| biomass00 | Unmatched | 136.89 | 123.60 | 41.90 |  | 0.30 |
|  | Matched | 136.89 | 136.41 | 1.50 | 96.40 | 0.01 |
| elev_m | Unmatched | 160.57 | 61.52 | 106.20 |  | 0.75 |
|  | Matched | 160.57 | 145.81 | 15.80 | 85.10 | 0.11 |
| forest00 | Unmatched | 93.00 | 90.16 | 19.80 |  | 0.14 |
|  | Matched | 93.00 | 93.49 | -3.40 | 82.70 | -0.02 |
| pop00 | Unmatched | 8.87 | 16.86 | -24.40 |  | -0.17 |
|  | Matched | 8.87 | 8.53 | 1.00 | 95.80 | 0.01 |
| slope_deg | Unmatched | 1.79 | 1.13 | 25.40 |  | 0.18 |
|  | Matched | 1.79 | 1.67 | 4.40 | 82.60 | 0.03 |
| precip | Unmatched | 3165.50 | 3191.10 | -8.00 |  | -0.06 |
|  | Matched | 3165.50 | 3134.90 | 9.60 | -19.50 | 0.07 |
