# Supplementary material for: The role of property rights in shaping the effectiveness of protected areas and resisting forest loss in the Yucatan Peninsula
Source: PLoS One. 2019 May 8;14(5):e0215820. doi: 10.1371/journal.pone.0215820 (PMC6505956; doi:10.1371/journal.pone.0215820)
Supplement: S5 Table — (DOCX) [file pone.0215820.s005.docx]

| **Variable** | **Sample** | **Mean** | | **%bias** | **%reduct  \|bias\|** | **norm. diff** |
| --- | --- | --- | --- | --- | --- | --- |
|  |  | **Treated** | **Control** |  |  |  |
| dist2inlandwater_km | Unmatched | 27.86 | 20.90 | 45.20 |  | 0.32 |
|  | Matched | 27.86 | 24.49 | 21.90 | 51.60 | 0.15 |
| dist2any_urban_km | Unmatched | 47.48 | 26.23 | 107.40 |  | 0.76 |
|  | Matched | 47.48 | 43.96 | 17.80 | 83.40 | 0.13 |
| dist2largefedrd_km | Unmatched | 38.58 | 20.41 | 91.20 |  | 0.64 |
|  | Matched | 38.58 | 36.51 | 10.40 | 88.60 | 0.07 |
| dist2largeurban_km | Unmatched | 132.23 | 99.80 | 70.40 |  | 0.50 |
|  | Matched | 132.23 | 122.56 | 21.00 | 70.20 | 0.15 |
| dist2pavedrd_km | Unmatched | 14.57 | 10.20 | 47.10 |  | 0.33 |
|  | Matched | 14.57 | 14.08 | 5.30 | 88.70 | 0.04 |
| dist2port_km | Unmatched | 180.07 | 175.69 | 7.60 |  | 0.05 |
|  | Matched | 180.07 | 180.73 | -1.10 | 85.00 | -0.01 |
| dist2unpavedrd_km | Unmatched | 33.84 | 17.01 | 112.90 |  | 0.80 |
|  | Matched | 33.84 | 30.28 | 23.90 | 78.90 | 0.17 |
| temper | Unmatched | 26.01 | 25.93 | 37.50 |  | 0.27 |
|  | Matched | 26.01 | 26.01 | 1.00 | 97.20 | 0.01 |
| biomass00 | Unmatched | 139.70 | 131.17 | 28.80 |  | 0.20 |
|  | Matched | 139.70 | 138.88 | 2.80 | 90.40 | 0.02 |
| elev_m | Unmatched | 161.24 | 66.47 | 115.30 |  | 0.82 |
|  | Matched | 161.24 | 152.04 | 11.20 | 90.30 | 0.08 |
| forest00 | Unmatched | 92.97 | 93.60 | -5.20 |  | -0.04 |
|  | Matched | 92.97 | 93.30 | -2.80 | 46.60 | -0.02 |
| pop00 | Unmatched | 7.97 | 15.71 | -24.30 |  | -0.17 |
|  | Matched | 7.97 | 7.88 | 0.30 | 98.90 | 0.00 |
| slope_deg | Unmatched | 1.97 | 1.27 | 28.00 |  | 0.20 |
|  | Matched | 1.97 | 2.00 | -0.90 | 96.70 | -0.01 |
| precip | Unmatched | 3089.00 | 3104.10 | -5.70 |  | -0.04 |
|  | Matched | 3089.00 | 3059.30 | 11.30 | -96.20 | 0.08 |
