# Supplementary material for: The role of property rights in shaping the effectiveness of protected areas and resisting forest loss in the Yucatan Peninsula
Source: PLoS One. 2019 May 8;14(5):e0215820. doi: 10.1371/journal.pone.0215820 (PMC6505956; doi:10.1371/journal.pone.0215820)
Supplement: S6 Table — (DOCX) [file pone.0215820.s006.docx]

| **Variable** | **Sample** | **Mean** | | **%bias** | **%reduct  \|bias\|** | **norm. diff** |
| --- | --- | --- | --- | --- | --- | --- |
|  |  | **Treated** | **Control** |  |  |  |
| dist2inlandwate | Unmatched | 18.13 | 44.11 | -133.10 |  | -0.94 |
|  | Matched | 18.13 | 22.61 | -22.90 | 82.80 | -0.16 |
| dist2any_urban_ | Unmatched | 18.41 | 10.86 | 72.70 |  | 0.51 |
|  | Matched | 18.41 | 15.78 | 25.30 | 65.20 | 0.18 |
| dist2largefedrd | Unmatched | 19.01 | 11.30 | 76.20 |  | 0.54 |
|  | Matched | 19.01 | 16.86 | 21.30 | 72.00 | 0.15 |
| dist2largeurban | Unmatched | 49.07 | 84.00 | -97.50 |  | -0.69 |
|  | Matched | 49.07 | 50.14 | -3.00 | 96.90 | -0.02 |
| dist2pavedrd_km | Unmatched | 6.61 | 4.80 | 44.50 |  | 0.31 |
|  | Matched | 6.61 | 5.72 | 21.90 | 50.70 | 0.15 |
| dist2port_km | Unmatched | 72.34 | 105.86 | -73.10 |  | -0.52 |
|  | Matched | 72.34 | 72.47 | -0.30 | 99.60 | 0.00 |
| dist2unpavedrd_ | Unmatched | 12.32 | 19.00 | -61.40 |  | -0.43 |
|  | Matched | 12.32 | 12.44 | -1.10 | 98.20 | -0.01 |
| temper | Unmatched | 26.00 | 25.91 | 42.40 |  | 0.30 |
|  | Matched | 26.00 | 25.99 | 7.70 | 81.70 | 0.05 |
| biomass00 | Unmatched | 108.57 | 105.85 | 9.10 |  | 0.06 |
|  | Matched | 108.57 | 106.74 | 6.10 | 32.50 | 0.04 |
| elev_m | Unmatched | 12.12 | 39.93 | -93.60 |  | -0.66 |
|  | Matched | 12.12 | 13.03 | -3.10 | 96.70 | -0.02 |
| forest00 | Unmatched | 81.15 | 82.66 | -8.20 |  | -0.06 |
|  | Matched | 81.15 | 80.10 | 5.70 | 31.10 | 0.04 |
| pop00 | Unmatched | 44.30 | 28.35 | 16.60 |  | 0.12 |
|  | Matched | 44.30 | 46.60 | -2.40 | 85.60 | -0.02 |
| slope_deg | Unmatched | 0.27 | 1.00 | -43.00 |  | -0.30 |
|  | Matched | 0.27 | 0.22 | 3.10 | 92.90 | 0.02 |
| precip | Unmatched | 3189.70 | 2905.30 | 125.60 |  | 0.89 |
|  | Matched | 3189.70 | 3129.40 | 26.60 | 78.80 | 0.19 |
