# Supplementary material for: The role of property rights in shaping the effectiveness of protected areas and resisting forest loss in the Yucatan Peninsula
Source: PLoS One. 2019 May 8;14(5):e0215820. doi: 10.1371/journal.pone.0215820 (PMC6505956; doi:10.1371/journal.pone.0215820)
Supplement: S7 Table — (DOCX) [file pone.0215820.s007.docx]

| Variable | Sample | Mean | | %bias | %reduct  \|bias\| | norm. diff |
| --- | --- | --- | --- | --- | --- | --- |
|  |  | Treated | Control |  |  |  |
| dist2inlandwate | Unmatched | 15.46 | 16.37 | -6.60 |  | -0.05 |
|  | Matched | 15.46 | 11.32 | 30.10 | -355.10 | 0.21 |
| dist2any_urban_ | Unmatched | 24.04 | 25.63 | -11.40 |  | -0.08 |
|  | Matched | 24.04 | 25.52 | -10.60 | 7.20 | -0.07 |
| dist2largefedrd | Unmatched | 25.90 | 11.13 | 129.10 |  | 0.91 |
|  | Matched | 25.90 | 21.98 | 34.20 | 73.50 | 0.24 |
| dist2largeurban | Unmatched | 158.52 | 125.10 | 41.10 |  | 0.29 |
|  | Matched | 158.52 | 154.50 | 4.90 | 88.00 | 0.03 |
| dist2pavedrd_km | Unmatched | 13.49 | 8.70 | 53.40 |  | 0.38 |
|  | Matched | 13.49 | 13.09 | 4.50 | 91.60 | 0.03 |
| dist2port_km | Unmatched | 94.59 | 101.85 | -12.90 |  | -0.09 |
|  | Matched | 94.59 | 102.14 | -13.40 | -4.00 | -0.09 |
| dist2unpavedrd_ | Unmatched | 19.13 | 12.92 | 71.20 |  | 0.50 |
|  | Matched | 19.13 | 16.88 | 25.80 | 63.80 | 0.18 |
| temper | Unmatched | 26.59 | 26.38 | 37.70 |  | 0.27 |
|  | Matched | 26.59 | 26.56 | 3.90 | 89.70 | 0.03 |
| biomass00 | Unmatched | 108.52 | 108.65 | -0.40 |  | 0.00 |
|  | Matched | 108.52 | 108.27 | 0.70 | -82.80 | 0.00 |
| elev_m | Unmatched | 54.93 | 24.96 | 37.20 |  | 0.26 |
|  | Matched | 54.93 | 43.78 | 13.80 | 62.80 | 0.10 |
| forest00 | Unmatched | 83.54 | 84.54 | -4.60 |  | -0.03 |
|  | Matched | 83.54 | 84.20 | -3.10 | 33.70 | -0.02 |
| pop00 | Unmatched | 7.00 | 21.84 | -33.40 |  | -0.24 |
|  | Matched | 7.00 | 5.45 | 3.50 | 89.60 | 0.02 |
| slope_deg | Unmatched | 0.36 | 0.53 | -11.60 |  | -0.08 |
|  | Matched | 0.36 | 0.27 | 6.70 | 42.30 | 0.05 |
| precip | Unmatched | 3346.80 | 3205.00 | 43.70 |  | 0.31 |
|  | Matched | 3346.80 | 3286.40 | 18.60 | 57.40 | 0.13 |
