# Supplementary material for: The role of property rights in shaping the effectiveness of protected areas and resisting forest loss in the Yucatan Peninsula
Source: PLoS One. 2019 May 8;14(5):e0215820. doi: 10.1371/journal.pone.0215820 (PMC6505956; doi:10.1371/journal.pone.0215820)
Supplement: S8 Table — (DOCX) [file pone.0215820.s008.docx]

| **Variable** | **Sample** | **Mean** | | **%bias** | **%reduct  \|bias\|** | **norm. diff** |
| --- | --- | --- | --- | --- | --- | --- |
|  |  | **Treated** | **Control** |  |  |  |
| dist2inlandwater_km | Unmatched | 13.765 | 14.644 | -5.7 |  | -0.04 |
|  | Matched | 13.765 | 12.163 | 10.3 | -82.3 | 0.07 |
| dist2any_urban_km | Unmatched | 22.657 | 23.495 | -5.8 |  | -0.04 |
|  | Matched | 22.657 | 20.861 | 12.3 | -114.2 | 0.09 |
| dist2largefedrd_km | Unmatched | 27.311 | 14.037 | 103.9 |  | 0.73 |
|  | Matched | 27.311 | 27.668 | -2.8 | 97.3 | -0.02 |
| dist2largeurban_km | Unmatched | 174.88 | 142.99 | 42.8 |  | 0.30 |
|  | Matched | 174.88 | 172.12 | 3.7 | 91.4 | 0.03 |
| dist2pavedrd_km | Unmatched | 15.215 | 8.7549 | 63.2 |  | 0.45 |
|  | Matched | 15.215 | 12.828 | 23.3 | 63 | 0.16 |
| dist2port_km | Unmatched | 102.48 | 106.25 | -7 |  | -0.05 |
|  | Matched | 102.48 | 90.845 | 21.6 | -209.3 | 0.15 |
| dist2unpavedrd_km | Unmatched | 14.239 | 11.991 | 23.1 |  | 0.16 |
|  | Matched | 14.239 | 11.502 | 28.2 | -21.7 | 0.20 |
| temper | Unmatched | 26.626 | 26.257 | 78.4 |  | 0.55 |
|  | Matched | 26.626 | 26.633 | -1.5 | 98.1 | -0.01 |
| biomass00 | Unmatched | 105.33 | 102.9 | 6.4 |  | 0.05 |
|  | Matched | 105.33 | 101.08 | 11.2 | -74.9 | 0.08 |
| elev_m | Unmatched | 68.434 | 44.371 | 26.6 |  | 0.19 |
|  | Matched | 68.434 | 63.324 | 5.7 | 78.8 | 0.04 |
| forest00 | Unmatched | 81.189 | 80.006 | 5.1 |  | 0.04 |
|  | Matched | 81.189 | 79.811 | 6 | -16.5 | 0.04 |
| pop00 | Unmatched | 10.3 | 17.04 | -20.1 |  | -0.14 |
|  | Matched | 10.3 | 10.823 | -1.6 | 92.2 | -0.01 |
| slope_deg | Unmatched | 0.48461 | 1.2756 | -37.1 |  | -0.26 |
|  | Matched | 0.48461 | 0.74059 | -12 | 67.6 | -0.08 |
| precip | Unmatched | 3532.1 | 3235.5 | 104.3 |  | 0.74 |
|  | Matched | 3532.1 | 3485.3 | 16.5 | 84.2 | 0.12 |
