# Supplementary material for: The role of property rights in shaping the effectiveness of protected areas and resisting forest loss in the Yucatan Peninsula
Source: PLoS One. 2019 May 8;14(5):e0215820. doi: 10.1371/journal.pone.0215820 (PMC6505956; doi:10.1371/journal.pone.0215820)
Supplement: S9 Table — (DOCX) [file pone.0215820.s009.docx]

| **Variable** | **Sample** | **Mean** | | **%bias** | **%reduct  \|bias\|** | **norm. diff** |
| --- | --- | --- | --- | --- | --- | --- |
|  |  | **Treated** | **Control** |  |  |  |
| dist2inlandwate | Unmatched | 39.07 | 40.96 | -9.80 |  | -0.07 |
|  | Matched | 39.07 | 36.01 | 15.90 | -61.90 | 0.11 |
| dist2any_urban_ | Unmatched | 18.74 | 12.40 | 50.70 |  | 0.36 |
|  | Matched | 18.74 | 20.27 | -12.30 | 75.80 | -0.09 |
| dist2largefedrd | Unmatched | 23.48 | 13.76 | 65.90 |  | 0.47 |
|  | Matched | 23.48 | 22.30 | 8.00 | 87.80 | 0.06 |
| dist2largeurban | Unmatched | 81.61 | 82.49 | -2.30 |  | -0.02 |
|  | Matched | 81.61 | 75.84 | 15.00 | -555.50 | 0.11 |
| dist2pavedrd_km | Unmatched | 8.97 | 5.26 | 63.40 |  | 0.45 |
|  | Matched | 8.97 | 8.62 | 6.00 | 90.50 | 0.04 |
| dist2port_km | Unmatched | 97.14 | 104.33 | -18.50 |  | -0.13 |
|  | Matched | 97.14 | 90.69 | 16.60 | 10.40 | 0.12 |
| dist2unpavedrd_ | Unmatched | 14.33 | 19.71 | -38.00 |  | -0.27 |
|  | Matched | 14.33 | 14.50 | -1.20 | 96.90 | -0.01 |
| temper | Unmatched | 26.00 | 25.92 | 44.70 |  | 0.32 |
|  | Matched | 26.00 | 26.01 | -4.40 | 90.30 | -0.03 |
| biomass00 | Unmatched | 109.83 | 101.51 | 24.30 |  | 0.17 |
|  | Matched | 109.83 | 104.20 | 16.40 | 32.30 | 0.12 |
| elev_m | Unmatched | 73.19 | 39.28 | 69.50 |  | 0.49 |
|  | Matched | 73.19 | 71.60 | 3.30 | 95.30 | 0.02 |
| forest00 | Unmatched | 80.51 | 79.98 | 2.60 |  | 0.02 |
|  | Matched | 80.51 | 77.08 | 16.90 | -550.50 | 0.12 |
| pop00 | Unmatched | 32.51 | 33.39 | -0.90 |  | -0.01 |
|  | Matched | 32.51 | 40.18 | -7.70 | -766.20 | -0.05 |
| slope_deg | Unmatched | 1.30 | 0.95 | 15.40 |  | 0.11 |
|  | Matched | 1.30 | 1.54 | -10.80 | 29.40 | -0.08 |
| precip | Unmatched | 2832.30 | 2891.00 | -41.00 |  | -0.29 |
|  | Matched | 2832.30 | 2839.90 | -5.30 | 87.00 | -0.04 |
