# Supplementary material for: The role of property rights in shaping the effectiveness of protected areas and resisting forest loss in the Yucatan Peninsula
Source: PLoS One. 2019 May 8;14(5):e0215820. doi: 10.1371/journal.pone.0215820 (PMC6505956; doi:10.1371/journal.pone.0215820)
Supplement: S10 Table — (DOCX) [file pone.0215820.s010.docx]

| **Variable** | **Sample** | **Mean** | | %bias | %reduct  \|bias\| | norm. diff |
| --- | --- | --- | --- | --- | --- | --- |
|  |  | **Treated** | **Control** |  |  |  |
| dist2inlandwate | Unmatched | 20.06 | 18.33 | 13.80 |  | 0.10 |
|  | Matched | 20.06 | 19.55 | 4.10 | 70.60 | 0.03 |
| dist2any_urban_ | Unmatched | 36.42 | 27.47 | 50.70 |  | 0.36 |
|  | Matched | 36.42 | 33.90 | 14.30 | 71.90 | 0.10 |
| dist2largefedrd | Unmatched | 29.58 | 21.58 | 42.10 |  | 0.30 |
|  | Matched | 29.58 | 28.22 | 7.20 | 83.00 | 0.05 |
| dist2largeurban | Unmatched | 110.12 | 106.94 | 6.10 |  | 0.04 |
|  | Matched | 110.12 | 109.99 | 0.20 | 96.20 | 0.00 |
| dist2pavedrd_km | Unmatched | 14.65 | 10.73 | 42.50 |  | 0.30 |
|  | Matched | 14.65 | 13.24 | 15.30 | 64.10 | 0.11 |
| dist2port_km | Unmatched | 153.29 | 154.80 | -2.40 |  | -0.02 |
|  | Matched | 153.29 | 154.05 | -1.20 | 49.70 | -0.01 |
| dist2unpavedrd_ | Unmatched | 21.66 | 17.46 | 32.00 |  | 0.23 |
|  | Matched | 21.66 | 20.66 | 7.60 | 76.20 | 0.05 |
| temper | Unmatched | 25.93 | 26.04 | -44.30 |  | -0.31 |
|  | Matched | 25.93 | 25.93 | -1.20 | 97.30 | -0.01 |
| biomass00 | Unmatched | 130.84 | 123.11 | 24.90 |  | 0.18 |
|  | Matched | 130.84 | 131.50 | -2.10 | 91.50 | -0.01 |
| elev_m | Unmatched | 86.86 | 57.79 | 42.40 |  | 0.30 |
|  | Matched | 86.86 | 84.90 | 2.90 | 93.20 | 0.02 |
| forest00 | Unmatched | 93.82 | 90.17 | 25.20 |  | 0.18 |
|  | Matched | 93.82 | 93.95 | -0.90 | 96.50 | -0.01 |
| pop00 | Unmatched | 7.57 | 16.90 | -28.00 |  | -0.20 |
|  | Matched | 7.57 | 7.26 | 0.90 | 96.70 | 0.01 |
| slope_deg | Unmatched | 1.32 | 1.12 | 8.50 |  | 0.06 |
|  | Matched | 1.32 | 1.21 | 4.50 | 46.90 | 0.03 |
| precip | Unmatched | 3048.00 | 3177.50 | -52.80 |  | -0.37 |
|  | Matched | 3048.00 | 3044.90 | 1.30 | 97.50 | 0.01 |
