# Supplementary material for: The role of property rights in shaping the effectiveness of protected areas and resisting forest loss in the Yucatan Peninsula
Source: PLoS One. 2019 May 8;14(5):e0215820. doi: 10.1371/journal.pone.0215820 (PMC6505956; doi:10.1371/journal.pone.0215820)
Supplement: S11 Table — (DOCX) [file pone.0215820.s011.docx]

| **Variable** | **Sample** | **Mean** | | **%bias** | **%reduct  \|bias\|** | **norm. diff** |
| --- | --- | --- | --- | --- | --- | --- |
|  |  | **Treated** | **Control** |  |  |  |
| dist2inlandwate | Unmatched | 24.45 | 40.86 | -82.40 |  | -0.58 |
|  | Matched | 24.45 | 28.24 | -19.00 | 76.90 | -0.13 |
| dist2any_urban_ | Unmatched | 16.82 | 12.83 | 29.40 |  | 0.21 |
|  | Matched | 16.82 | 17.79 | -7.10 | 75.90 | -0.05 |
| dist2largefedrd | Unmatched | 17.01 | 14.42 | 19.60 |  | 0.14 |
|  | Matched | 17.01 | 17.33 | -2.40 | 87.70 | -0.02 |
| dist2largeurban | Unmatched | 86.88 | 82.42 | 11.30 |  | 0.08 |
|  | Matched | 86.88 | 92.08 | -13.10 | -16.50 | -0.09 |
| dist2pavedrd_km | Unmatched | 5.17 | 5.51 | -7.10 |  | -0.05 |
|  | Matched | 5.17 | 4.79 | 7.80 | -10.50 | 0.06 |
| dist2port_km | Unmatched | 108.25 | 103.83 | 9.90 |  | 0.07 |
|  | Matched | 108.25 | 115.41 | -16.00 | -62.10 | -0.11 |
| dist2unpavedrd_ | Unmatched | 19.16 | 19.34 | -1.30 |  | -0.01 |
|  | Matched | 19.16 | 22.05 | -20.80 | -1461.20 | -0.15 |
| temper | Unmatched | 25.86 | 25.92 | -26.90 |  | -0.19 |
|  | Matched | 25.86 | 25.90 | -15.10 | 43.80 | -0.11 |
| biomass00 | Unmatched | 94.39 | 102.09 | -24.30 |  | -0.17 |
|  | Matched | 94.39 | 96.30 | -6.00 | 75.20 | -0.04 |
| elev_m | Unmatched | 13.40 | 41.64 | -92.80 |  | -0.66 |
|  | Matched | 13.40 | 12.58 | 2.70 | 97.10 | 0.02 |
| forest00 | Unmatched | 79.56 | 80.02 | -2.10 |  | -0.01 |
|  | Matched | 79.56 | 82.57 | -14.00 | -551.00 | -0.10 |
| pop00 | Unmatched | 39.01 | 33.32 | 6.20 |  | 0.04 |
|  | Matched | 39.01 | 35.84 | 3.50 | 44.20 | 0.02 |
| slope_deg | Unmatched | 0.15 | 0.97 | -51.80 |  | -0.37 |
|  | Matched | 0.15 | 0.20 | -3.00 | 94.20 | -0.02 |
| precip | Unmatched | 3000.10 | 2886.80 | 66.80 |  | 0.47 |
|  | Matched | 3000.10 | 3027.90 | -16.40 | 75.50 | -0.12 |
