# Supplementary material for: The role of property rights in shaping the effectiveness of protected areas and resisting forest loss in the Yucatan Peninsula
Source: PLoS One. 2019 May 8;14(5):e0215820. doi: 10.1371/journal.pone.0215820 (PMC6505956; doi:10.1371/journal.pone.0215820)
Supplement: S12 Table — (DOCX) [file pone.0215820.s012.docx]

| **Variable** | **Sample** | **Mean** | | **%bias** | **%reduct  \|bias\|** | **norm. diff** |
| --- | --- | --- | --- | --- | --- | --- |
|  |  | **Treated** | **Control** |  |  |  |
| dist2inlandwater_km | Unmatched | 15.43 | 18.62 | -28.30 |  | -0.20 |
|  | Matched | 15.43 | 15.33 | 0.90 | 96.70 | 0.01 |
| dist2any_urban_km | Unmatched | 28.20 | 28.90 | -4.50 |  | -0.03 |
|  | Matched | 28.20 | 27.85 | 2.20 | 49.80 | 0.02 |
| dist2largefedrd_km | Unmatched | 21.60 | 22.86 | -7.80 |  | -0.06 |
|  | Matched | 21.60 | 21.45 | 0.90 | 88.40 | 0.01 |
| dist2largeurban_km | Unmatched | 116.70 | 107.40 | 12.70 |  | 0.09 |
|  | Matched | 116.70 | 124.81 | -11.00 | 12.90 | -0.08 |
| dist2pavedrd_km | Unmatched | 17.41 | 11.32 | 70.30 |  | 0.50 |
|  | Matched | 17.41 | 16.64 | 8.90 | 87.30 | 0.06 |
| dist2port_km | Unmatched | 82.90 | 154.92 | -126.30 |  | -0.89 |
|  | Matched | 82.90 | 86.24 | -5.90 | 95.40 | -0.04 |
| dist2unpavedrd_km | Unmatched | 13.59 | 18.15 | -43.50 |  | -0.31 |
|  | Matched | 13.59 | 13.68 | -0.90 | 97.90 | -0.01 |
| temper | Unmatched | 26.41 | 26.02 | 91.00 |  | 0.64 |
|  | Matched | 26.41 | 26.45 | -9.50 | 89.60 | -0.07 |
| biomass00 | Unmatched | 104.30 | 124.44 | -63.00 |  | -0.45 |
|  | Matched | 104.30 | 104.88 | -1.80 | 97.10 | -0.01 |
| elev_m | Unmatched | 12.84 | 62.99 | -102.30 |  | -0.72 |
|  | Matched | 12.84 | 13.77 | -1.90 | 98.10 | -0.01 |
| forest00 | Unmatched | 85.89 | 90.77 | -25.70 |  | -0.18 |
|  | Matched | 85.89 | 84.43 | 7.70 | 70.00 | 0.05 |
| pop00 | Unmatched | 6.31 | 15.42 | -29.80 |  | -0.21 |
|  | Matched | 6.31 | 6.24 | 0.20 | 99.20 | 0.00 |
| slope_deg | Unmatched | 0.23 | 1.16 | -53.20 |  | -0.38 |
|  | Matched | 0.23 | 0.23 | 0.30 | 99.40 | 0.00 |
| precip | Unmatched | 3278.60 | 3156.30 | 34.60 |  | 0.24 |
|  | Matched | 3278.60 | 3322.10 | -12.30 | 64.50 | -0.09 |
