# Supplementary material for: The role of property rights in shaping the effectiveness of protected areas and resisting forest loss in the Yucatan Peninsula
Source: PLoS One. 2019 May 8;14(5):e0215820. doi: 10.1371/journal.pone.0215820 (PMC6505956; doi:10.1371/journal.pone.0215820)
Supplement: S13 Table — (DOCX) [file pone.0215820.s013.docx]

| **Variable** | **Sample** | **Mean** | | **%bias** | **%reduct  \|bias\|** | **norm. diff** |
| --- | --- | --- | --- | --- | --- | --- |
|  |  | **Treated** | **Control** |  |  |  |
| dist2inlandwate | Unmatched | 24.65 | 41.12 | -90.80 |  | -0.64 |
|  | Matched | 24.65 | 24.77 | -0.70 | 99.30 | 0.00 |
| dist2any_urban_ | Unmatched | 9.68 | 12.70 | -29.00 |  | -0.21 |
|  | Matched | 9.68 | 10.61 | -9.00 | 69.20 | -0.06 |
| dist2largefedrd | Unmatched | 13.58 | 14.56 | -6.90 |  | -0.05 |
|  | Matched | 13.58 | 13.87 | -2.10 | 70.10 | -0.01 |
| dist2largeurban | Unmatched | 41.97 | 80.78 | -114.10 |  | -0.81 |
|  | Matched | 41.97 | 44.19 | -6.50 | 94.30 | -0.05 |
| dist2pavedrd_km | Unmatched | 4.07 | 5.39 | -30.70 |  | -0.22 |
|  | Matched | 4.07 | 4.79 | -16.70 | 45.60 | -0.12 |
| dist2port_km | Unmatched | 69.95 | 103.61 | -92.20 |  | -0.65 |
|  | Matched | 69.95 | 70.70 | -2.10 | 97.80 | -0.01 |
| dist2unpavedrd_ | Unmatched | 22.43 | 19.02 | 22.70 |  | 0.16 |
|  | Matched | 22.43 | 21.59 | 5.60 | 75.30 | 0.04 |
| temper | Unmatched | 25.99 | 25.93 | 29.10 |  | 0.21 |
|  | Matched | 25.99 | 26.01 | -10.90 | 62.40 | -0.08 |
| biomass00 | Unmatched | 86.48 | 102.79 | -48.10 |  | -0.34 |
|  | Matched | 86.48 | 88.47 | -5.90 | 87.80 | -0.04 |
| elev_m | Unmatched | 23.03 | 43.43 | -53.00 |  | -0.37 |
|  | Matched | 23.03 | 24.67 | -4.30 | 91.90 | -0.03 |
| forest00 | Unmatched | 70.58 | 80.03 | -44.80 |  | -0.32 |
|  | Matched | 70.58 | 72.13 | -7.40 | 83.60 | -0.05 |
| pop00 | Unmatched | 128.71 | 32.49 | 54.00 |  | 0.38 |
|  | Matched | 128.71 | 109.72 | 10.70 | 80.30 | 0.08 |
| slope_deg | Unmatched | 0.77 | 1.04 | -12.20 |  | -0.09 |
|  | Matched | 0.77 | 0.73 | 1.80 | 85.00 | 0.01 |
| precip | Unmatched | 2852.50 | 2879.40 | -15.20 |  | -0.11 |
|  | Matched | 2852.50 | 2865.90 | -7.50 | 50.30 | -0.05 |
