# Supplementary material for: The role of property rights in shaping the effectiveness of protected areas and resisting forest loss in the Yucatan Peninsula
Source: PLoS One. 2019 May 8;14(5):e0215820. doi: 10.1371/journal.pone.0215820 (PMC6505956; doi:10.1371/journal.pone.0215820)
Supplement: S14 Table — (DOCX) [file pone.0215820.s014.docx]

| **Variable** | **Sample** | **Mean** | | **%bias** | **%reduct  \|bias\|** | **norm. diff** |
| --- | --- | --- | --- | --- | --- | --- |
|  |  | **Treated** | **Control** |  |  |  |
| dist2inlandwater_km | Unmatched | 16.89 | 18.64 | -16.10 |  | -0.11 |
|  | Matched | 16.89 | 16.82 | 0.70 | 95.90 | 0.00 |
| dist2any_urban_km | Unmatched | 24.49 | 28.98 | -23.50 |  | -0.17 |
|  | Matched | 24.49 | 24.29 | 1.00 | 95.60 | 0.01 |
| dist2largefedrd_km | Unmatched | 19.55 | 22.91 | -20.20 |  | -0.14 |
|  | Matched | 19.55 | 20.83 | -7.70 | 61.90 | -0.05 |
| dist2largeurban_km | Unmatched | 97.80 | 107.64 | -17.50 |  | -0.12 |
|  | Matched | 97.80 | 98.44 | -1.10 | 93.50 | -0.01 |
| dist2pavedrd_km | Unmatched | 7.32 | 11.43 | -52.40 |  | -0.37 |
|  | Matched | 7.32 | 7.76 | -5.70 | 89.20 | -0.04 |
| dist2port_km | Unmatched | 116.14 | 155.33 | -69.00 |  | -0.49 |
|  | Matched | 116.14 | 118.09 | -3.40 | 95.00 | -0.02 |
| dist2unpavedrd_km | Unmatched | 23.67 | 18.01 | 40.80 |  | 0.29 |
|  | Matched | 23.67 | 24.05 | -2.70 | 93.30 | -0.02 |
| temper | Unmatched | 26.06 | 26.02 | 18.60 |  | 0.13 |
|  | Matched | 26.06 | 26.05 | 5.20 | 71.90 | 0.04 |
| biomass00 | Unmatched | 114.83 | 124.53 | -30.40 |  | -0.21 |
|  | Matched | 114.83 | 116.51 | -5.30 | 82.60 | -0.04 |
| elev_m | Unmatched | 65.77 | 62.66 | 5.20 |  | 0.04 |
|  | Matched | 65.77 | 66.11 | -0.60 | 89.30 | 0.00 |
| forest00 | Unmatched | 89.33 | 90.78 | -9.00 |  | -0.06 |
|  | Matched | 89.33 | 89.77 | -2.70 | 69.70 | -0.02 |
| pop00 | Unmatched | 13.62 | 15.41 | -4.70 |  | -0.03 |
|  | Matched | 13.62 | 12.91 | 1.90 | 60.40 | 0.01 |
| slope_deg | Unmatched | 1.41 | 1.15 | 11.10 |  | 0.08 |
|  | Matched | 1.41 | 1.38 | 1.50 | 86.60 | 0.01 |
| precip | Unmatched | 3050.60 | 3159.00 | -45.20 |  | -0.32 |
|  | Matched | 3050.60 | 3055.30 | -2.00 | 95.60 | -0.01 |
