# Supplementary material for: The role of property rights in shaping the effectiveness of protected areas and resisting forest loss in the Yucatan Peninsula
Source: PLoS One. 2019 May 8;14(5):e0215820. doi: 10.1371/journal.pone.0215820 (PMC6505956; doi:10.1371/journal.pone.0215820)
Supplement: S15 Table — (DOCX) [file pone.0215820.s015.docx]

| **Variable** | **Sample** | **Mean** | | **%bias** | **%reduct  \|bias\|** | **norm. diff** |
| --- | --- | --- | --- | --- | --- | --- |
|  |  | **Treated** | **Control** |  |  |  |
| dist2inlandwater_km | Unmatched | 12.631 | 13.27 | -5.4 |  | -0.04 |
|  | Matched | 12.631 | 10.952 | 14.2 | -162.8 | 0.10 |
| dist2any_urban_km | Unmatched | 24.081 | 23.251 | 6.1 |  | 0.04 |
|  | Matched | 24.081 | 25.72 | -12 | -97.4 | -0.08 |
| dist2largefedrd_km | Unmatched | 28.292 | 25.531 | 22.1 |  | 0.16 |
|  | Matched | 28.292 | 27.727 | 4.5 | 79.5 | 0.03 |
| dist2largeurban_km | Unmatched | 44.564 | 51.828 | -21.4 |  | -0.15 |
|  | Matched | 44.564 | 43.721 | 2.5 | 88.4 | 0.02 |
| dist2pavedrd_km | Unmatched | 11.42 | 10.093 | 16.9 |  | 0.12 |
|  | Matched | 11.42 | 13.474 | -26.2 | -54.7 | -0.19 |
| dist2port_km | Unmatched | 90.374 | 85.046 | 13.8 |  | 0.10 |
|  | Matched | 90.374 | 90.63 | -0.7 | 95.2 | 0.00 |
| dist2unpavedrd_km | Unmatched | 19.376 | 18.669 | 6.5 |  | 0.05 |
|  | Matched | 19.376 | 16.529 | 26.1 | -302.9 | 0.18 |
| temper | Unmatched | 25.939 | 25.899 | 18.7 |  | 0.13 |
|  | Matched | 25.939 | 25.945 | -3.2 | 82.7 | -0.02 |
| biomass00 | Unmatched | 95.32 | 95.94 | -2.2 |  | -0.02 |
|  | Matched | 95.32 | 95.49 | -0.6 | 72.6 | 0.00 |
| elev_m | Unmatched | 7.8088 | 7.7575 | 0.4 |  | 0.00 |
|  | Matched | 7.8088 | 7.8779 | -0.6 | -34.6 | 0.00 |
| forest00 | Unmatched | 83.374 | 79.105 | 20.7 |  | 0.15 |
|  | Matched | 83.374 | 84.375 | -4.9 | 76.6 | -0.03 |
| pop00 | Unmatched | 105.72 | 94.383 | 4.8 |  | 0.03 |
|  | Matched | 105.72 | 107.04 | -0.6 | 88.4 | 0.00 |
| slope_deg | Unmatched | 0.16574 | 0.17317 | -1.1 |  | -0.01 |
|  | Matched | 0.16574 | 0.15397 | 1.7 | -58.5 | 0.01 |
| precip | Unmatched | 2960.7 | 3035.6 | -33.4 |  | -0.24 |
|  | Matched | 2960.7 | 2976.4 | -7 | 79.1 | -0.05 |
