# Supplementary material for: The role of property rights in shaping the effectiveness of protected areas and resisting forest loss in the Yucatan Peninsula
Source: PLoS One. 2019 May 8;14(5):e0215820. doi: 10.1371/journal.pone.0215820 (PMC6505956; doi:10.1371/journal.pone.0215820)
Supplement: S16 Table — (DOCX) [file pone.0215820.s016.docx]

| **Variable** | **Sample** | **Mean** | | **%bias** | **%reduct  \|bias\|** | **norm. diff** |
| --- | --- | --- | --- | --- | --- | --- |
|  |  | **Treated** | **Control** |  |  |  |
| dist2inlandwate | Unmatched | 28.90 | 30.21 | -7.00 |  | -0.05 |
|  | Matched | 28.90 | 26.58 | 12.30 | -76.50 | 0.09 |
| dist2any_urban_ | Unmatched | 43.38 | 51.20 | -37.90 |  | -0.27 |
|  | Matched | 43.38 | 41.34 | 9.90 | 73.90 | 0.07 |
| dist2largefedrd | Unmatched | 37.09 | 42.28 | -24.60 |  | -0.17 |
|  | Matched | 37.09 | 35.15 | 9.20 | 62.60 | 0.07 |
| dist2largeurban | Unmatched | 125.91 | 141.60 | -33.50 |  | -0.24 |
|  | Matched | 125.91 | 118.92 | 14.90 | 55.50 | 0.11 |
| dist2pavedrd_km | Unmatched | 13.63 | 19.78 | -56.40 |  | -0.40 |
|  | Matched | 13.63 | 13.64 | -0.20 | 99.70 | 0.00 |
| dist2port_km | Unmatched | 176.80 | 181.96 | -9.60 |  | -0.07 |
|  | Matched | 176.80 | 166.77 | 18.70 | -94.20 | 0.13 |
| dist2unpavedrd_ | Unmatched | 30.83 | 31.45 | -3.80 |  | -0.03 |
|  | Matched | 30.83 | 27.82 | 18.50 | -388.20 | 0.13 |
| temper | Unmatched | 26.04 | 26.05 | -3.50 |  | -0.02 |
|  | Matched | 26.04 | 26.07 | -11.00 | -217.50 | -0.08 |
| biomass00 | Unmatched | 137.42 | 135.98 | 4.70 |  | 0.03 |
|  | Matched | 137.42 | 132.26 | 17.00 | -258.60 | 0.12 |
| elev_m | Unmatched | 155.99 | 171.14 | -14.10 |  | -0.10 |
|  | Matched | 155.99 | 133.71 | 20.80 | -47.00 | 0.15 |
| forest00 | Unmatched | 92.60 | 92.33 | 2.20 |  | 0.02 |
|  | Matched | 92.60 | 92.77 | -1.40 | 38.70 | -0.01 |
| pop00 | Unmatched | 7.80 | 9.87 | -43.70 |  | -0.31 |
|  | Matched | 7.80 | 7.34 | 9.70 | 77.90 | 0.07 |
| slope_deg | Unmatched | 1.77 | 1.79 | -0.60 |  | 0.00 |
|  | Matched | 1.77 | 1.35 | 16.10 | -2662.80 | 0.11 |
| precip | Unmatched | 3073.20 | 3230.70 | -48.10 |  | -0.34 |
|  | Matched | 3073.20 | 3043.40 | 9.10 | 81.10 | 0.06 |
