# Supplementary material for: The role of property rights in shaping the effectiveness of protected areas and resisting forest loss in the Yucatan Peninsula
Source: PLoS One. 2019 May 8;14(5):e0215820. doi: 10.1371/journal.pone.0215820 (PMC6505956; doi:10.1371/journal.pone.0215820)
Supplement: S17 Table — (DOCX) [file pone.0215820.s017.docx]

| **Variable** | **Sample** | **Mean** | | **%bias** | **%reduct  \|bias\|** | **norm. diff** |
| --- | --- | --- | --- | --- | --- | --- |
|  |  | **Treated** | **Control** |  |  |  |
| dist2inlandwate | Unmatched | 9.33 | 13.51 | -43.60 |  | -0.31 |
|  | Matched | 9.33 | 11.57 | -23.30 | 46.40 | -0.16 |
| dist2any_urban_ | Unmatched | 6.90 | 25.84 | -192.70 |  | -1.36 |
|  | Matched | 6.90 | 7.04 | -1.40 | 99.30 | -0.01 |
| dist2largefedrd | Unmatched | 12.79 | 28.51 | -167.50 |  | -1.18 |
|  | Matched | 12.79 | 12.38 | 4.40 | 97.40 | 0.03 |
| dist2largeurban | Unmatched | 19.43 | 52.92 | -109.00 |  | -0.77 |
|  | Matched | 19.43 | 19.04 | 1.30 | 98.80 | 0.01 |
| dist2pavedrd_km | Unmatched | 2.59 | 11.71 | -154.70 |  | -1.09 |
|  | Matched | 2.59 | 2.50 | 1.60 | 99.00 | 0.01 |
| dist2port_km | Unmatched | 53.65 | 91.71 | -116.60 |  | -0.82 |
|  | Matched | 53.65 | 53.71 | -0.20 | 99.90 | 0.00 |
| dist2unpavedrd_ | Unmatched | 13.75 | 19.66 | -52.90 |  | -0.37 |
|  | Matched | 13.75 | 12.67 | 9.60 | 81.80 | 0.07 |
| temper | Unmatched | 25.98 | 25.91 | 40.50 |  | 0.29 |
|  | Matched | 25.98 | 25.96 | 10.40 | 74.30 | 0.07 |
| biomass00 | Unmatched | 81.57 | 97.60 | -58.60 |  | -0.41 |
|  | Matched | 81.57 | 80.30 | 4.70 | 92.00 | 0.03 |
| elev_m | Unmatched | 6.94 | 7.89 | -9.80 |  | -0.07 |
|  | Matched | 6.94 | 7.20 | -2.70 | 72.10 | -0.02 |
| forest00 | Unmatched | 68.83 | 82.43 | -68.10 |  | -0.48 |
|  | Matched | 68.83 | 69.28 | -2.20 | 96.70 | -0.02 |
| pop00 | Unmatched | 371.84 | 61.98 | 109.20 |  | 0.77 |
|  | Matched | 371.84 | 399.13 | -9.60 | 91.20 | -0.07 |
| slope_deg | Unmatched | 0.09 | 0.18 | -15.30 |  | -0.11 |
|  | Matched | 0.09 | 0.02 | 12.50 | 18.10 | 0.09 |
| precip | Unmatched | 2962.00 | 3011.60 | -19.60 |  | -0.14 |
|  | Matched | 2962.00 | 2952.20 | 3.90 | 80.10 | 0.03 |
