# Supplementary material for: The role of property rights in shaping the effectiveness of protected areas and resisting forest loss in the Yucatan Peninsula
Source: PLoS One. 2019 May 8;14(5):e0215820. doi: 10.1371/journal.pone.0215820 (PMC6505956; doi:10.1371/journal.pone.0215820)
Supplement: S18 Table — (DOCX) [file pone.0215820.s018.docx]

| **Variable** | **Sample** | **Mean** | | **%bias** | **%reduct  \|bias\|** | **norm. diff** |
| --- | --- | --- | --- | --- | --- | --- |
|  |  | **Treated** | **Control** |  |  |  |
| dist2inlandwater_km | Unmatched | 30.05 | 29.98 | 0.30 |  | 0.00 |
|  | Matched | 30.05 | 33.01 | -15.20 | -4343.60 | -0.11 |
| dist2any_urban_km | Unmatched | 41.01 | 51.24 | -48.80 |  | -0.35 |
|  | Matched | 41.01 | 38.10 | 13.80 | 71.60 | 0.10 |
| dist2largefedrd_km | Unmatched | 33.17 | 42.66 | -44.50 |  | -0.31 |
|  | Matched | 33.17 | 32.55 | 2.90 | 93.50 | 0.02 |
| dist2largeurban_km | Unmatched | 130.09 | 140.34 | -21.20 |  | -0.15 |
|  | Matched | 130.09 | 129.34 | 1.50 | 92.70 | 0.01 |
| dist2pavedrd_km | Unmatched | 13.32 | 19.58 | -58.90 |  | -0.42 |
|  | Matched | 13.32 | 13.03 | 2.80 | 95.30 | 0.02 |
| dist2port_km | Unmatched | 178.93 | 181.43 | -4.50 |  | -0.03 |
|  | Matched | 178.93 | 173.48 | 9.80 | -118.60 | 0.07 |
| dist2unpavedrd_km | Unmatched | 27.86 | 31.87 | -24.40 |  | -0.17 |
|  | Matched | 27.86 | 24.25 | 22.00 | 10.00 | 0.16 |
| temper | Unmatched | 26.05 | 26.04 | 1.20 |  | 0.01 |
|  | Matched | 26.05 | 26.07 | -8.30 | -592.70 | -0.06 |
| biomass00 | Unmatched | 136.89 | 136.12 | 2.50 |  | 0.02 |
|  | Matched | 136.89 | 134.05 | 9.10 | -268.00 | 0.06 |
| elev_m | Unmatched | 160.24 | 169.84 | -8.70 |  | -0.06 |
|  | Matched | 160.24 | 167.47 | -6.50 | 24.70 | -0.05 |
| forest00 | Unmatched | 91.93 | 92.45 | -4.10 |  | -0.03 |
|  | Matched | 91.93 | 90.62 | 10.40 | -154.20 | 0.07 |
| pop00 | Unmatched | 8.68 | 9.66 | -21.00 |  | -0.15 |
|  | Matched | 8.68 | 7.76 | 19.80 | 5.50 | 0.14 |
| slope_deg | Unmatched | 1.70 | 1.80 | -3.60 |  | -0.03 |
|  | Matched | 1.70 | 1.50 | 7.60 | -108.90 | 0.05 |
| precip | Unmatched | 3136.40 | 3214.90 | -23.30 |  | -0.16 |
|  | Matched | 3136.40 | 3127.40 | 2.70 | 88.50 | 0.02 |
