# Supplementary material for: The role of property rights in shaping the effectiveness of protected areas and resisting forest loss in the Yucatan Peninsula
Source: PLoS One. 2019 May 8;14(5):e0215820. doi: 10.1371/journal.pone.0215820 (PMC6505956; doi:10.1371/journal.pone.0215820)
Supplement: S19 Table — Because of the small control pool (n = 32), matching was not possible. (DOCX) [file pone.0215820.s019.docx]

| **Variable** | **Mean** | | **%bias** | **norm. diff** |
| --- | --- | --- | --- | --- |
|  | **Treated** | **Control** |  |  |
| dist2inlandwater_km | 16.52 | 11.34 | 35.40 | 0.25 |
| dist2any_urban_km | 23.20 | 16.36 | 46.70 | 0.33 |
| dist2largefedrd_km | 23.50 | 21.98 | 12.60 | 0.09 |
| dist2largeurban_km | 44.54 | 55.27 | -23.40 | -0.17 |
| dist2pavedrd_km | 12.02 | 4.61 | 106.10 | 0.75 |
| dist2port_km | 78.85 | 91.04 | -27.40 | -0.19 |
| dist2unpavedrd_km | 14.34 | 19.60 | -38.30 | -0.27 |
| temper | 25.98 | 25.76 | 70.00 | 0.49 |
| biomass00 | 109.64 | 67.72 | 184.70 | 1.31 |
| elev_m | 10.46 | 7.91 | 18.70 | 0.13 |
| forest00 | 85.79 | 65.88 | 101.90 | 0.72 |
| pop00 | 32.10 | 389.29 | -126.10 | -0.89 |
| slope_deg | 0.24 | 0.08 | 22.30 | 0.16 |
| precip | 3116.50 | 3002.80 | 47.40 | 0.34 |
