# Supplementary material for: The role of property rights in shaping the effectiveness of protected areas and resisting forest loss in the Yucatan Peninsula
Source: PLoS One. 2019 May 8;14(5):e0215820. doi: 10.1371/journal.pone.0215820 (PMC6505956; doi:10.1371/journal.pone.0215820)
Supplement: S20 Table — (DOCX) [file pone.0215820.s020.docx]

| **Variable** | **Sample** | **Mean** | | **%bias** | **%reduct  \|bias\|** | **norm. diff** |
| --- | --- | --- | --- | --- | --- | --- |
|  |  | **Treated** | **Control** |  |  |  |
| dist2inlandwater_km | Unmatched | 15.23 | 30.28 | -83.50 |  | -0.59 |
|  | Matched | 15.23 | 14.62 | 3.40 | 96.00 | 0.02 |
| dist2any_urban_km | Unmatched | 21.28 | 50.47 | -173.70 |  | -1.23 |
|  | Matched | 21.28 | 20.49 | 4.70 | 97.30 | 0.03 |
| dist2largefedrd_km | Unmatched | 24.59 | 41.75 | -107.50 |  | -0.76 |
|  | Matched | 24.59 | 24.52 | 0.40 | 99.60 | 0.00 |
| dist2largeurban_km | Unmatched | 166.05 | 138.46 | 40.90 |  | 0.29 |
|  | Matched | 166.05 | 182.14 | -23.90 | 41.70 | -0.17 |
| dist2pavedrd_km | Unmatched | 12.72 | 18.88 | -54.90 |  | -0.39 |
|  | Matched | 12.72 | 10.88 | 16.40 | 70.20 | 0.12 |
| dist2port_km | Unmatched | 89.53 | 182.92 | -174.50 |  | -1.23 |
|  | Matched | 89.53 | 97.07 | -14.10 | 91.90 | -0.10 |
| dist2unpavedrd_km | Unmatched | 18.33 | 31.60 | -100.50 |  | -0.71 |
|  | Matched | 18.33 | 18.99 | -5.00 | 95.00 | -0.04 |
| temper | Unmatched | 26.65 | 26.03 | 131.20 |  | 0.93 |
|  | Matched | 26.65 | 26.72 | -15.90 | 87.90 | -0.11 |
| biomass00 | Unmatched | 106.12 | 136.82 | -96.10 |  | -0.68 |
|  | Matched | 106.12 | 105.11 | 3.20 | 96.70 | 0.02 |
| elev_m | Unmatched | 52.12 | 170.93 | -108.70 |  | -0.77 |
|  | Matched | 52.12 | 51.39 | 0.70 | 99.40 | 0.00 |
| forest00 | Unmatched | 81.97 | 92.59 | -59.20 |  | -0.42 |
|  | Matched | 81.97 | 79.95 | 11.20 | 81.00 | 0.08 |
| pop00 | Unmatched | 7.03 | 9.58 | -55.60 |  | -0.39 |
|  | Matched | 7.03 | 6.91 | 2.70 | 95.20 | 0.02 |
| slope_deg | Unmatched | 0.34 | 1.81 | -70.40 |  | -0.50 |
|  | Matched | 0.34 | 0.39 | -2.60 | 96.30 | -0.02 |
| precip | Unmatched | 3382.90 | 3201.00 | 50.50 |  | 0.36 |
|  | Matched | 3382.90 | 3463.60 | -22.40 | 55.60 | -0.16 |
