# Supplementary material for: The role of property rights in shaping the effectiveness of protected areas and resisting forest loss in the Yucatan Peninsula
Source: PLoS One. 2019 May 8;14(5):e0215820. doi: 10.1371/journal.pone.0215820 (PMC6505956; doi:10.1371/journal.pone.0215820)
Supplement: S21 Table — (DOCX) [file pone.0215820.s021.docx]

| **Variable** | **Sample** | **Mean** | | **%bias** | **%reduct  \|bias\|** | **norm. diff** |
| --- | --- | --- | --- | --- | --- | --- |
|  |  | **Treated** | **Control** |  |  |  |
| dist2inlandwater_km | Unmatched | 41.69 | 40.68 | 5.60 |  | 0.04 |
|  | Matched | 41.69 | 42.23 | -3.00 | 46.40 | -0.02 |
| dist2any_urban_km | Unmatched | 11.22 | 12.95 | -19.60 |  | -0.14 |
|  | Matched | 11.22 | 10.76 | 5.20 | 73.60 | 0.04 |
| dist2largefedrd_km | Unmatched | 13.06 | 13.83 | -7.20 |  | -0.05 |
|  | Matched | 13.06 | 12.35 | 6.60 | 8.90 | 0.05 |
| dist2largeurban_km | Unmatched | 83.62 | 82.47 | 3.10 |  | 0.02 |
|  | Matched | 83.62 | 83.53 | 0.20 | 92.10 | 0.00 |
| dist2pavedrd_km | Unmatched | 4.78 | 5.42 | -15.50 |  | -0.11 |
|  | Matched | 4.78 | 4.69 | 2.10 | 86.20 | 0.01 |
| dist2port_km | Unmatched | 106.80 | 103.45 | 8.00 |  | 0.06 |
|  | Matched | 106.80 | 106.84 | -0.10 | 98.80 | 0.00 |
| dist2unpavedrd_km | Unmatched | 18.88 | 20.39 | -9.80 |  | -0.07 |
|  | Matched | 18.88 | 19.10 | -1.40 | 85.40 | -0.01 |
| temper | Unmatched | 25.94 | 25.90 | 17.20 |  | 0.12 |
|  | Matched | 25.94 | 25.94 | 0.80 | 95.10 | 0.01 |
| biomass00 | Unmatched | 100.30 | 102.20 | -5.80 |  | -0.04 |
|  | Matched | 100.30 | 100.54 | -0.70 | 87.20 | 0.00 |
| elev_m | Unmatched | 33.60 | 42.06 | -22.50 |  | -0.16 |
|  | Matched | 33.60 | 33.00 | 1.60 | 92.90 | 0.01 |
| forest00 | Unmatched | 80.11 | 79.99 | 0.60 |  | 0.00 |
|  | Matched | 80.11 | 80.39 | -1.40 | -141.50 | -0.01 |
| pop00 | Unmatched | 32.12 | 33.26 | -1.30 |  | -0.01 |
|  | Matched | 32.12 | 32.36 | -0.30 | 78.90 | 0.00 |
| slope_deg | Unmatched | 0.84 | 1.01 | -7.60 |  | -0.05 |
|  | Matched | 0.84 | 0.80 | 2.10 | 71.90 | 0.01 |
| precip | Unmatched | 2902.80 | 2885.40 | 11.20 |  | 0.08 |
|  | Matched | 2902.80 | 2901.00 | 1.10 | 90.00 | 0.01 |
