# Supplementary material for: The role of property rights in shaping the effectiveness of protected areas and resisting forest loss in the Yucatan Peninsula
Source: PLoS One. 2019 May 8;14(5):e0215820. doi: 10.1371/journal.pone.0215820 (PMC6505956; doi:10.1371/journal.pone.0215820)
Supplement: S22 Table — (DOCX) [file pone.0215820.s022.docx]

| **Variable** | **Sample** | **Mean** | | **%bias** | **%reduct  \|bias\|** | **norm. diff** |
| --- | --- | --- | --- | --- | --- | --- |
|  |  | **Treated** | **Control** |  |  |  |
| dist2inlandwater_km | Unmatched | 20.81 | 15.61 | 46.60 |  | 0.33 |
|  | Matched | 20.81 | 19.86 | 8.50 | 81.70 | 0.06 |
| dist2any_urban_km | Unmatched | 24.68 | 29.83 | -31.70 |  | -0.22 |
|  | Matched | 24.68 | 25.44 | -4.70 | 85.20 | -0.03 |
| dist2largefedrd_km | Unmatched | 18.55 | 24.04 | -30.90 |  | -0.22 |
|  | Matched | 18.55 | 17.19 | 7.60 | 75.30 | 0.05 |
| dist2largeurban_km | Unmatched | 97.17 | 117.46 | -33.50 |  | -0.24 |
|  | Matched | 97.17 | 98.06 | -1.50 | 95.60 | -0.01 |
| dist2pavedrd_km | Unmatched | 10.28 | 10.81 | -6.20 |  | -0.04 |
|  | Matched | 10.28 | 8.99 | 15.10 | -143.30 | 0.11 |
| dist2port_km | Unmatched | 172.56 | 139.03 | 45.50 |  | 0.32 |
|  | Matched | 172.56 | 171.32 | 1.70 | 96.30 | 0.01 |
| dist2unpavedrd_km | Unmatched | 15.93 | 18.33 | -18.50 |  | -0.13 |
|  | Matched | 15.93 | 17.48 | -11.90 | 35.70 | -0.08 |
| temper | Unmatched | 25.93 | 26.16 | -77.10 |  | -0.55 |
|  | Matched | 25.93 | 25.92 | 1.70 | 97.80 | 0.01 |
| biomass00 | Unmatched | 130.10 | 115.56 | 44.20 |  | 0.31 |
|  | Matched | 130.10 | 129.90 | 0.60 | 98.60 | 0.00 |
| elev_m | Unmatched | 59.94 | 50.68 | 16.40 |  | 0.12 |
|  | Matched | 59.94 | 64.82 | -8.60 | 47.40 | -0.06 |
| forest00 | Unmatched | 93.64 | 86.35 | 42.80 |  | 0.30 |
|  | Matched | 93.64 | 92.18 | 8.50 | 80.00 | 0.06 |
| pop00 | Unmatched | 15.94 | 18.60 | -5.60 |  | -0.04 |
|  | Matched | 15.94 | 14.21 | 3.70 | 34.60 | 0.03 |
| slope_deg | Unmatched | 1.16 | 1.04 | 5.60 |  | 0.04 |
|  | Matched | 1.16 | 1.38 | -9.40 | -68.40 | -0.07 |
| precip | Unmatched | 3088.10 | 3288.60 | -72.70 |  | -0.51 |
|  | Matched | 3088.10 | 3091.00 | -1.10 | 98.60 | -0.01 |
