# Supplementary material for: The role of property rights in shaping the effectiveness of protected areas and resisting forest loss in the Yucatan Peninsula
Source: PLoS One. 2019 May 8;14(5):e0215820. doi: 10.1371/journal.pone.0215820 (PMC6505956; doi:10.1371/journal.pone.0215820)
Supplement: S23 Table — (DOCX) [file pone.0215820.s023.docx]

| **Variable** | **Sample** | **Mean** | | **%bias** | **%reduct  \|bias\|** | **norm. diff** |
| --- | --- | --- | --- | --- | --- | --- |
|  |  | **Treated** | **Control** |  |  |  |
| dist2inlandwater_km | Unmatched | 40.89 | 41.19 | -1.70 |  | -0.01 |
|  | Matched | 40.89 | 38.62 | 12.70 | -649.90 | 0.09 |
| dist2any_urban_km | Unmatched | 11.55 | 12.74 | -13.40 |  | -0.09 |
|  | Matched | 11.55 | 11.29 | 2.90 | 78.50 | 0.02 |
| dist2largefedrd_km | Unmatched | 13.33 | 13.66 | -3.00 |  | -0.02 |
|  | Matched | 13.33 | 12.31 | 9.40 | -213.90 | 0.07 |
| dist2largeurban_km | Unmatched | 83.53 | 82.53 | 2.70 |  | 0.02 |
|  | Matched | 83.53 | 78.29 | 13.90 | -422.50 | 0.10 |
| dist2pavedrd_km | Unmatched | 4.78 | 5.42 | -15.50 |  | -0.11 |
|  | Matched | 4.78 | 4.46 | 7.70 | 50.60 | 0.05 |
| dist2port_km | Unmatched | 106.94 | 103.36 | 8.60 |  | 0.06 |
|  | Matched | 106.94 | 101.11 | 14.00 | -62.80 | 0.10 |
| dist2unpavedrd_km | Unmatched | 19.06 | 20.27 | -7.90 |  | -0.06 |
|  | Matched | 19.06 | 18.17 | 5.80 | 26.70 | 0.04 |
| temper | Unmatched | 25.94 | 25.90 | 16.20 |  | 0.11 |
|  | Matched | 25.94 | 25.95 | -3.90 | 75.70 | -0.03 |
| biomass00 | Unmatched | 100.12 | 102.32 | -6.80 |  | -0.05 |
|  | Matched | 100.12 | 97.38 | 8.40 | -24.20 | 0.06 |
| elev_m | Unmatched | 33.35 | 42.22 | -23.60 |  | -0.17 |
|  | Matched | 33.35 | 31.62 | 4.60 | 80.60 | 0.03 |
| forest00 | Unmatched | 80.02 | 80.04 | -0.10 |  | 0.00 |
|  | Matched | 80.02 | 78.38 | 8.20 | -7618.60 | 0.06 |
| pop00 | Unmatched | 32.06 | 33.30 | -1.40 |  | -0.01 |
|  | Matched | 32.06 | 34.49 | -2.70 | -96.70 | -0.02 |
| slope_deg | Unmatched | 0.84 | 1.01 | -7.50 |  | -0.05 |
|  | Matched | 0.84 | 0.92 | -3.80 | 50.00 | -0.03 |
| precip | Unmatched | 2903.10 | 2885.20 | 11.60 |  | 0.08 |
|  | Matched | 2903.10 | 2918.80 | -10.20 | 12.60 | -0.07 |
