# Supplementary material for: The role of property rights in shaping the effectiveness of protected areas and resisting forest loss in the Yucatan Peninsula
Source: PLoS One. 2019 May 8;14(5):e0215820. doi: 10.1371/journal.pone.0215820 (PMC6505956; doi:10.1371/journal.pone.0215820)
Supplement: S24 Table — (DOCX) [file pone.0215820.s024.docx]

| **Variable** | **Sample** | **Mean** | | **%bias** | **%reduct  \|bias\|** | **norm. diff** |
| --- | --- | --- | --- | --- | --- | --- |
|  |  | **Treated** | **Control** |  |  |  |
| dist2inlandwate | Unmatched | 20.72 | 15.71 | 44.90 |  | 0.32 |
|  | Matched | 20.72 | 20.94 | -1.90 | 95.70 | -0.01 |
| dist2any_urban_ | Unmatched | 24.34 | 30.20 | -36.10 |  | -0.26 |
|  | Matched | 24.34 | 24.77 | -2.70 | 92.60 | -0.02 |
| dist2largefedrd | Unmatched | 19.05 | 23.48 | -24.90 |  | -0.18 |
|  | Matched | 19.05 | 19.08 | -0.20 | 99.30 | 0.00 |
| dist2largeurban | Unmatched | 98.70 | 115.76 | -28.10 |  | -0.20 |
|  | Matched | 98.70 | 98.39 | 0.50 | 98.20 | 0.00 |
| dist2pavedrd_km | Unmatched | 9.37 | 11.82 | -29.00 |  | -0.21 |
|  | Matched | 9.37 | 9.50 | -1.60 | 94.50 | -0.01 |
| dist2port_km | Unmatched | 173.50 | 138.00 | 48.40 |  | 0.34 |
|  | Matched | 173.50 | 174.21 | -1.00 | 98.00 | -0.01 |
| dist2unpavedrd_ | Unmatched | 15.96 | 18.30 | -18.10 |  | -0.13 |
|  | Matched | 15.96 | 16.23 | -2.10 | 88.50 | -0.01 |
| temper | Unmatched | 25.93 | 26.15 | -74.40 |  | -0.53 |
|  | Matched | 25.93 | 25.94 | -2.90 | 96.10 | -0.02 |
| biomass00 | Unmatched | 130.73 | 114.88 | 48.50 |  | 0.34 |
|  | Matched | 130.73 | 126.13 | 14.00 | 71.00 | 0.10 |
| elev_m | Unmatched | 62.86 | 46.80 | 29.00 |  | 0.21 |
|  | Matched | 62.86 | 60.18 | 4.80 | 83.30 | 0.03 |
| forest00 | Unmatched | 93.46 | 86.54 | 40.50 |  | 0.29 |
|  | Matched | 93.46 | 92.07 | 8.20 | 79.80 | 0.06 |
| pop00 | Unmatched | 15.77 | 18.81 | -6.40 |  | -0.05 |
|  | Matched | 15.77 | 13.50 | 4.80 | 25.10 | 0.03 |
| slope_deg | Unmatched | 1.22 | 0.96 | 11.60 |  | 0.08 |
|  | Matched | 1.22 | 1.36 | -6.20 | 47.00 | -0.04 |
| precip | Unmatched | 3106.00 | 3268.80 | -57.90 |  | -0.41 |
|  | Matched | 3106.00 | 3101.40 | 1.60 | 97.20 | 0.01 |
