# Supplementary material for: The role of property rights in shaping the effectiveness of protected areas and resisting forest loss in the Yucatan Peninsula
Source: PLoS One. 2019 May 8;14(5):e0215820. doi: 10.1371/journal.pone.0215820 (PMC6505956; doi:10.1371/journal.pone.0215820)
Supplement: S25 Table — (DOCX) [file pone.0215820.s025.docx]

| **Variable** | **Sample** | **Mean** | | **%bias** | **%reduct  \|bias\|** | **norm. diff** |
| --- | --- | --- | --- | --- | --- | --- |
|  |  | **Treated** | **Control** |  |  |  |
| dist2inlandwate | Unmatched | 42.59 | 40.60 | 11.20 |  | 0.08 |
|  | Matched | 42.59 | 37.92 | 26.30 | -134.00 | 0.19 |
| dist2any_urban_ | Unmatched | 10.68 | 12.78 | -23.70 |  | -0.17 |
|  | Matched | 10.68 | 11.42 | -8.40 | 64.50 | -0.06 |
| dist2largefedrd | Unmatched | 10.96 | 14.34 | -33.00 |  | -0.23 |
|  | Matched | 10.96 | 10.83 | 1.20 | 96.30 | 0.01 |
| dist2largeurban | Unmatched | 84.22 | 82.51 | 4.60 |  | 0.03 |
|  | Matched | 84.22 | 84.24 | -0.10 | 98.50 | 0.00 |
| dist2pavedrd_km | Unmatched | 4.68 | 5.32 | -15.90 |  | -0.11 |
|  | Matched | 4.68 | 4.42 | 6.30 | 60.20 | 0.04 |
| dist2port_km | Unmatched | 106.28 | 104.28 | 4.80 |  | 0.03 |
|  | Matched | 106.28 | 106.61 | -0.80 | 83.30 | -0.01 |
| dist2unpavedrd_ | Unmatched | 20.56 | 19.56 | 6.70 |  | 0.05 |
|  | Matched | 20.56 | 17.59 | 19.90 | -196.90 | 0.14 |
| temper | Unmatched | 25.89 | 25.92 | -13.00 |  | -0.09 |
|  | Matched | 25.89 | 25.95 | -23.10 | -77.60 | -0.16 |
| biomass00 | Unmatched | 103.27 | 100.88 | 7.30 |  | 0.05 |
|  | Matched | 103.27 | 101.63 | 5.00 | 31.60 | 0.04 |
| elev_m | Unmatched | 37.36 | 39.19 | -4.90 |  | -0.03 |
|  | Matched | 37.36 | 35.81 | 4.20 | 14.80 | 0.03 |
| forest00 | Unmatched | 81.65 | 79.53 | 10.60 |  | 0.07 |
|  | Matched | 81.65 | 80.60 | 5.20 | 50.70 | 0.04 |
| pop00 | Unmatched | 30.61 | 33.51 | -3.60 |  | -0.03 |
|  | Matched | 30.61 | 32.78 | -2.70 | 25.30 | -0.02 |
| slope_deg | Unmatched | 0.99 | 0.93 | 3.00 |  | 0.02 |
|  | Matched | 0.99 | 1.22 | -10.80 | -263.30 | -0.08 |
| precip | Unmatched | 2905.80 | 2887.90 | 11.60 |  | 0.08 |
|  | Matched | 2905.80 | 2945.30 | -25.50 | -119.60 | -0.18 |
