# Supplementary material for: The role of property rights in shaping the effectiveness of protected areas and resisting forest loss in the Yucatan Peninsula
Source: PLoS One. 2019 May 8;14(5):e0215820. doi: 10.1371/journal.pone.0215820 (PMC6505956; doi:10.1371/journal.pone.0215820)
Supplement: S26 Table — (DOCX) [file pone.0215820.s026.docx]

| **Variable** | **Sample** | **Mean** | | **%bias** | **%reduct  \|bias\|** | **norm. diff** |
| --- | --- | --- | --- | --- | --- | --- |
|  |  | **Treated** | **Control** |  |  |  |
| dist2inlandwate | Unmatched | 16.81 | 18.50 | -16.00 |  | -0.11 |
|  | Matched | 16.81 | 17.77 | -9.00 | 43.50 | -0.06 |
| dist2any_urban_ | Unmatched | 25.07 | 27.34 | -15.00 |  | -0.11 |
|  | Matched | 25.07 | 24.18 | 5.80 | 61.10 | 0.04 |
| dist2largefedrd | Unmatched | 11.18 | 22.22 | -76.40 |  | -0.54 |
|  | Matched | 11.18 | 13.29 | -14.60 | 80.90 | -0.10 |
| dist2largeurban | Unmatched | 127.35 | 104.63 | 33.20 |  | 0.23 |
|  | Matched | 127.35 | 127.56 | -0.30 | 99.10 | 0.00 |
| dist2pavedrd_km | Unmatched | 8.78 | 10.72 | -25.30 |  | -0.18 |
|  | Matched | 8.78 | 10.03 | -16.20 | 35.70 | -0.11 |
| dist2port_km | Unmatched | 96.84 | 162.98 | -103.90 |  | -0.73 |
|  | Matched | 96.84 | 101.61 | -7.50 | 92.80 | -0.05 |
| dist2unpavedrd_ | Unmatched | 12.51 | 17.56 | -44.80 |  | -0.32 |
|  | Matched | 12.51 | 13.21 | -6.20 | 86.10 | -0.04 |
| temper | Unmatched | 26.38 | 26.00 | 97.80 |  | 0.69 |
|  | Matched | 26.38 | 26.38 | -0.50 | 99.50 | 0.00 |
| biomass00 | Unmatched | 106.88 | 124.93 | -54.60 |  | -0.39 |
|  | Matched | 106.88 | 106.03 | 2.50 | 95.30 | 0.02 |
| elev_m | Unmatched | 26.51 | 59.40 | -67.30 |  | -0.48 |
|  | Matched | 26.51 | 33.77 | -14.90 | 77.90 | -0.11 |
| forest00 | Unmatched | 83.64 | 90.87 | -37.30 |  | -0.26 |
|  | Matched | 83.64 | 82.88 | 3.90 | 89.50 | 0.03 |
| pop00 | Unmatched | 21.46 | 16.68 | 8.80 |  | 0.06 |
|  | Matched | 21.46 | 19.91 | 2.90 | 67.50 | 0.02 |
| slope_deg | Unmatched | 0.59 | 1.17 | -27.70 |  | -0.20 |
|  | Matched | 0.59 | 0.63 | -2.00 | 92.70 | -0.01 |
| precip | Unmatched | 3201.30 | 3181.40 | 7.20 |  | 0.05 |
|  | Matched | 3201.30 | 3226.40 | -9.10 | -26.60 | -0.06 |
